# Overexpression of *ScMYBAS1* alternative splicing transcripts differentially impacts biomass accumulation and drought tolerance in rice transgenic plants

**DOI:** 10.1371/journal.pone.0207534

**Published:** 2018-12-05

**Authors:** Rafael Fávero Peixoto-Junior, Larissa Mara de Andrade, Michael dos Santos Brito, Paula Macedo Nobile, Alexandre Palma Boer Martins, Samira Domingues Carlin, Rafael Vasconcelos Ribeiro, Maria Helena de Souza Goldman, João Felipe Nebó Carlos de Oliveira, Antonio Vargas de Oliveira Figueira, Silvana Creste

**Affiliations:** 1 Departamento de Genética, Faculdade de Medicina de Ribeirão Preto, Universidade de São Paulo, Ribeirão Preto, SP, Brazil; 2 Centro de Cana, Instituto Agronômico, Ribeirão Preto, SP, Brazil; 3 Dept. Plant Biology, Institute of Biology, University of Campinas, Campinas, SP, Brazil; 4 Departamento de Biologia, Faculdade de Filosofia, Ciências e Letras de Ribeirão Preto, Universidade de São Paulo, Ribeirão Preto, SP, Brazil; 5 Centro de Energia Nuclear na Agricultura, Universidade de São Paulo, Piracicaba, SP, Brazil; Institute of Genetics and Developmental Biology Chinese Academy of Sciences, CHINA

## Abstract

Drought is the most significant environmental stress for agricultural production worldwide, and tremendous efforts have been made to improve crop yield under the increasing water scarcity. Transcription factors are major players in the regulation of water stress-related genes in plants. Recently, different MYB transcription factors were characterized for their involvement in drought response. A sugarcane R2R3-MYB gene (*ScMYBAS1*) and its four alternative forms of transcript (*ScMYAS1-2*, *ScMYBAS1-3*, *ScMYBAS1-4* and *ScMYBAS1-5*) were identified in this study. The subcellular localization, in *Nicotiniana benthamiana*, of the TFs fused in frame with GFP revealed that ScMYBAS1-2-GFP and ScMYBAS1-3-GFP were observed in the nucleus. The overexpression of *ScMYBAS1-2* and *ScMYBAS1-3* spliced transcripts in rice promoted change in plant growth under both well-watered and drought conditions. The ScMYBAS1-2 and ScMYBAS1-3 transgenic lines revealed a higher relative water content (RWC) compared to the wild type before maximum stress under drought conditions. The ScMYBAS1-2 transgenic lines showed a reduction in biomass (total dry weight). Conversely, ScMYBAS1-3 showed an increased biomass (total dry weight) relative to the wild-type. The overexpression of *ScMYBAS1-3* in rice transgenic lines showed involvement with drought tolerance and biomass and, for this reason, was considered a good target for plant transformation, particularly for use in developing genotypes with drought tolerance and biomass accumulation.

## Introduction

Plants are often exposed to various environmental stresses, such as drought, extreme temperatures and high salinity. These abiotic stresses dramatically impact the crop yield and growth. Among these stresses, drought is the main factor that negatively influences sugarcane crop productivity [1]. One of the strategies to overcome this external factor involves generating genotypes with an increased tolerance to drought using advanced molecular breeding techniques and biotechnological approaches. Several efforts have been made to identify and manipulate candidate genes to obtain drought-tolerant plants [2].

Transcription factors (TFs) regulate most aspects of the plant life cycle by regulating the expression of specific genes, including stress-responsive genes [3]. Several TFs are involved in the response of plants to drought and often participate in gene activation cascades, which act together to increase drought tolerance [4]. TFs are grouped into large families, such as **A**petala2 (AP2)/**E**thylene **R**esponsive **F**actor (ERF), **B**asic Leucine **Zip**per (bZIP), **N**o **A**pical **M**eristem (NAM), **A**rabidopsis **T**ranscription **A**ctivation **F**actor (ATAF), CUP/SHAPED Cotyledon (CUC), WRKY and **My**elo**b**lastosis (MYB), according to their protein structures and DNA binding sites [5]. Different MYB transcription factors have been characterized for their involvement in a variety of biological functions, including drought stress regulation [6]. Two distinct regions are found in MYB proteins: a non-conserved C-terminal modulator region and a N-terminal conserved MYB DNA-binding domain, which is responsible for the regulatory activity of the protein. The MYB DNA-binding domain has one to four imperfect repeats composed of 50–53 amino acids each and forms a helix-turn-helix fold with three regulatory spaced tryptophan residues [7]. Based on the number of imperfect repeats in the MYB DNA-binding domains, the MYB family is classified into four subfamilies: the 1R-, R2R3-, 3R- and 4R- MYB proteins [7, 8]. All four subfamilies are found in plants, so they represent the taxon with the highest diversity of MYB proteins. The 1R-MYB subfamily, also called the MYB-related group, is a heterogeneous group that contains proteins with an intact or partial repeat (such as R3 or R1/2). It is the second largest subfamily of MYB, and functional studies have demonstrated that they regulate plant development and stress responses [9]. The R2R3-MYB proteins are specific to plants and are the most abundant type in plants, with more than 100 R2R3-MYB members in the genomes of dicots and monocots [10, 11]. Over the past decade, the R2R3-MYB genes have been extensively studied, and members of this MYB family were found to be involved in regulating responses to drought, salt and cold [12]. The 3R-MYB (R1R2R3-MYB) subfamily is an evolutionarily conserved group in plants, whose members contain three repeats, each of which is similar to the vertebrate MYB repeats [13]. In contrast to the predominant roles of the 3R-MYB group in vertebrates, relatively few 3R-MYB proteins are present among the plant MYB proteins [14]. The smallest subfamily is 4R-MYB, whose members contain four R1/R2-like repeats. A single 4R-MYB protein is encoded in several plant genomes; for example, *Arabidopsis* has only one representative (At3g18100) in this class [12].

Recently, some MYB TFs in *Arabidopsis* and other plants were identified as being involved in drought responses [15]. The overexpression of *AtMYB96* improves drought tolerance either by integrating ABA and auxin signals [16] or by activating cuticular wax biosynthesis [17]. *AtMYB41* is induced by drought and may function as a transcription factor that modulates cell expansion and cuticle deposition during drought stress [18]. *AtMYB44*, *AtMYB60* and *AtMYB61* improve drought tolerance by regulating stomatal movement [19] [20, 21]. *AtMYB96*, *AtMYB15* and *AtMYB2*, induced by drought, act as positive regulators of drought tolerance by activating the transcription of dehydration responsive genes, such as *RD22* [16, 22–24]. Arabidopsis transgenic plants that overexpress *AtMYB15* exhibit hypersensitivity to exogenous ABA and show improved tolerance to drought [24] and cold stress [25]. Rice transgenic plants that overexpress *OsMYB2* exhibit enhanced tolerance to various stresses, conferred by a change in the expression levels of numerous genes involved in diverse functions in stress response [26]. The expression of *OsMYB4* and *OsMYB3R-2* is induced by drought in rice, and their ectopic expression promotes drought tolerance [27, 28]. The overexpression of sugarcane ScMYB18 in tobacco results in plants with a markedly improved tolerance to drought and salt stress [29]. The stress-related MYB TF, *ScMYB****AS****1* (**A**lternative **S**plicing), which is a R2R3-MYB subfamily, is induced in response to water deficiency and salt stress in sugarcane seedlings of Co740 [30]. Another study, also carried out with the TF *ScMYBAS1* but renamed as *ScMYB2* (GenBank Accession Number KM387411), revealed its involvement in the leaf senescence signalling pathway and showed that it plays a positive role in response to drought-induced senescence in sugarcane. In addition, two alternatively spliced transcripts were identified: *ScMYB2S1* and *ScMYB2S2* [31].

In a previous experiment carried out by our team, a microarray data analysis using the RNA from the sugarcane leaves of two Brazilian sugarcane genotypes, with a differential response to drought, revealed several classes of TFs that were up- or downregulated under drought conditions [32]. Among these TFs, *ScMYBAS1* was upregulated in response to drought. Therefore, based on previous evidence [33], we investigated in depth the involvement of *ScMYBAS1* in response to drought stress. Here, we report the functional characterization of two alternatively spliced forms of *ScMYBAS1* (*ScMYBAS1-2* and *ScMYBAS1-3*) in rice transgenic lines. *ScMYBAS1-2* and *ScMYBAS1-3* overexpression promoted a higher relative water content (RWC) in the rice transgenic lines under drought conditions compared with the wild-type. Interestingly, the ScMYBAS1-3 transgenic lines showed an increase in biomass, while the ScMYBAS1-2 transgenic lines showed a reduction in biomass, both compared with the wild-type plants.

## Materials and methods

### Plant materials

The sugarcane genotypes IACSP94-2094 and IACSP97-7065, developed by the “Centro de Cana, Instituto Agronômico”—IAC, Brazil), were used to isolate the full-length coding DNA sequence (CDS) of the *Sc**MYBAS1* gene (*S**a**c**charum* spp.). These genotypes have a differential growth and yield in drought-prone areas, with 'IACSP94-2094' being more tolerant to low water availability than 'IACSP97-7065'. Both genotypes were grown under field conditions (Goiás, Brazil, 15°13’S; 48°56’), and at 6-months-old, they were grown under no rainfall for 3 months. The leaves (+1) from the stressed and non-stressed plants were used for RNA extraction and gene cloning. For more details about the field trials, see Andrade et al, 2016. Rice seeds (*Oryza sativa* L. ssp. *japonica* cv. Nipponbare) were used to generate the rice transgenic plants. The wild-type (WT) and rice transgenic plants were grown under greenhouse conditions.

### Gene cloning, DNA sequencing and Phylogenetic analysis

The full-length CDS of *ScMYBAS1* was accessed in Sugarcane Expressed Sequence Tag (SUCEST) [34] and the National Center for Biotechnology Information (NCBI). The primers were designed flanking the initiation codon ‘ATG’ (ScMYBAS1_CDS_F) and the stop codon ‘TGA’ (ScMYBAS1_CDS_R) for the amplification of the complete coding sequence (CDS) using template cDNAs from genotypes IACSP94-2094 and IACSP97-7065 (Table 1). The cloning was performed using pGEM-T Easy (Promega, Fitchburg, WI, USA) and the *E*. *coli* strain DH10b according to the manufacturer’s instructions. The clones obtained were sequenced, and the chromatograms generated by the 3730/3730xl Data Collection Software v3.0 program (Applied Biosystems, Foster City, CA, USA) were analysed by the DNA Baser Sequence Assembler v3.2.5 program (http://www.dnabaser.com/). The ‘FASTA’ files were obtained for each clone individually, forming contigs containing only the sequences of interest and with good quality. The consensus DNA sequences that were obtained by overlapping the forward and reverse sequences for each clone were aligned using the ClustalX 2.0 program [35] and were visualized in the BioEdit program [36].

**Table 1:** Information and characteristics of the primers used in CDS (Coding Domain Sequence) amplification of *ScMYBAS1* for functional analysis; insertion of Gateway sites; analysis of expression in transgenic plants; confirmation of transgenics; and estimate the copy number of the transgene.

Primers	Access N°	Sequence (5´-3´)	A (bp)	T*m* (°C)	Reference
ScMYBAS1_CDS_F	SCSBAD1085B04	ATTATGGTGACTGTGAGGGAGGAG	670	58	*
ScMYBAS1_CDS_R		TCACATCATGATTTCTTTATCTTCCA			
*attB1ScMYBAS1*	SCSBAD1085B04	GCAGGCTTCACCACCATGGTGACTGTG	686	58	*
*attB2ScMYBAS1*		AAGCTGGGTCTCACATCATA			
*BP1*	-	GGGGACAAGTTTGTACAAAAAAGCAGGCTTC	-	57	[44]
*BP2*	-	GGGGACCACTTTGTACAAGAAAGCTGGGTC	-	59	[44]
*hpt II_ PCR_F*	pHb7m24GW^a^	GTGTATTGACCGATTCCTTG	240	55	*
*hpt II_PCR_R*		CGTTATGTTTATCGGCAGTT			
*p35S_PCR_F*	pHb7m24GW^a^	CGCAATGATGGCATTTGTAG	597	55	*
*p35S_PCR_R*		GCTGACCCACAGATGGTTAGA			
*eEF1a_qPCR_F*	AK061464	AAGAACGGTGATGCTGGTATG	88	60	[45]
*eEF1a_qPCR_R*		AACGACCAAGAGGAGGGTACT			
*ScMYBAS1_qPCR2_F*	SCSBAD1085B04	ACTGACATACCAAGCCTGCC	75	60	*
*ScMYBAS1_qPCR2_R*		CCACCATTGTGAGTGTCACC			
*hpt II_Taqman_F*	pHb7m24GW^a^	CGCAGCGATAGCATCCATAG	70	60	*
*hpt II_Taqman_R*		AGACCTGCCTGAAACCGAACT			
*SPS_Taqman_F*	U33175	TGCGCCTGAACGGATAT	81	60	[46]
*SPS_Taqman_R*		CGGTTGATCTTTTCGGGATG			
**Taqman Probe**					
*hptII_probe*	pHb7m24GW^a^	VIC-CCGGCTGAAGAAC-MGBNFQ			*
*SPS_probe*	U33175	6FAM-GACGCACGGACGGCTCGGA-MGBNFQ			[46]

A: amplicon in base pairs

Tm: Melting temperature

a Primes were drawn using a vector sequence as a template

* Primers developed in this work.

A dendrogram was constructed using the ScMYABS1 protein sequence for comparative search, using the Basic Local Alignment Search Tool (BLAST) [37] for monocot sequences with similarities above 70% in database sequences in the National Center for Biotechnology Information (NCBI), the Plant Genomics Resource (Phytozome v11) and the Grass Regulatory Information Server (GRASSIUS). The *Arabidopsis* sequences were obtained from The Arabidopsis Information Resource (TAIR). Multiple protein sequence alignments were performed using ClustalX 2.0 [35], while a phylogenetic tree was constructed by MEGA 7 [38], using a maximum parsimony method. A bootstrap analysis was performed using 100 replicates in MEGA 7 [39] to evaluate the reliability of the dendrogram.

### Subcellular localization

The full-length CDS of *ScMYBAS1*, without the stop codon ‘TGA’, was cloned into the pDONR221 (Invitrogen, Carlsbad, CA, USA) vector using the Gateway BP Clonase II Enzyme Mix (Invitrogen, Carlsbad, CA, USA). To generate a ScMYBAS1-GFP fusion protein, the pENTR (with the full-length CDS of *ScMYBAS1* without stop codon) was recombined with the pK7FWG2 vector (https://gateway.psb.ugent.be/search) using Gateway LR Clonase II Enzyme Mix (Invitrogen, Carlsbad, CA, USA) (Karime et al, 2007). The construct was used for transient transformation of the *Nicotiana benthamiana* leaves via *Agrobacterium* infiltration [40]. After an incubation for 3–5 days at 28°C, GFP fluorescence was observed under a Leica SP5 confocal microscope (Leica Microsystems, Wetzlar, DE). Leaves stained with 4',6-diamidino-2-phenylindole (DAPI) were used as a positive control of nuclei localization. *In silico* analyses, using MultiLoc online tools, were also employed for predicting the protein subcellular localization using the ScMYBAS1 sequence [41].

### Generation of transgenic rice plants

To produce transgenic rice plants overexpressing *ScMYBAS1*, the full-length CDS of *ScMYBAS1-2* and *ScMYBAS1-3* were amplified by PCR using primers with the attB1 (attB1ScMYBAS1) and attB2 (attB2ScMYBAS1) Gateway sites (Invitrogen, Carlsbad, CA, USA) (Table 1). The sequences were confirmed by sequencing and cloned into the pDONR221 (Invitrogen) vector using the Gateway BP Clonase II Enzyme Mix (Invitrogen, Carlsbad, CA, USA), resulting in pENTR vectors. The overexpression vectors were obtained using multiple recombination among the pENTR (with ScMYBAS1-2 and *ScMYBAS1-3*), pHb7m24GW and pEN-L4-UBIL vectors (https://gateway.psb.ugent.be/search) using the Gateway LR Clonase II Enzyme Mix (Invitrogen, Carlsbad, CA, USA) [42]. All the constructs were transformed into the *E*. *coli* strain DH10b and, after sequencing confirmation, the overexpression vectors were transformed into the *Agrobacterium tumefaciens* strain EHA-105. The *ScMYBAS1* overexpression vectors, driven by the maize Ubiquitin promoter (Ubi-1), were introduced into the rice via *Agrobacterium tumefaciens*-mediate transformation [43]. The *Hygromycin phosphotransferase* (*hpt* II) gene was used to screen for the positive rice transgenic plants. The transformed calli were placed on MS3 agar medium supplemented with 50 mg L^-1^ hygromycin (Sigma, Saint Louis, MO, USA) and were maintained at 26°C, with a photoperiod of 14/10 h (light/dark), with subcultures obtained every 15 days [43]. The T_0_ rice transgenic plants were confirmed by PCR using the 35S promoter (p35S_PCR_F and p35S_PCR_R) and the *hpt* II (hptII_ PCR_F and hptII_PCR_R) primers (Table 1).

### RNA isolation, analysis of gene expression and estimation of transgene copy number

The total RNA was isolated from the leaves with the TRIzol reagent (Life Technologies, Carlsbad, CA, USA) following manufacturer’s instructions. Genomic DNA contamination in the total RNA samples was eliminated by RNAse-free DNAse I (Promega). The total RNA was then used as a template for first-strand cDNA synthesis by Superscript II (Invitrogen). For the transgene expression analysis, quantitative real-time PCR was performed on a StepOnePlus Real-Time PCR System (Applied Biosystems, Foster City, CA, USA) with the SYBR green qPCR Master Mix (Applied Biosystems). The cycling conditions were 10 min at 95°C followed by 40 cycles of amplification (95°C for 15 s, 60°C for 30 s, and 72°C for 30 s). Rice *elongation factor 1 alpha* (*eEF1a*) (primers eEF1a_qPCR_F and eEF1a_qPCR_R, Table 1) was used as an endogenous control for normalization [45], and the target gene was *ScMYBSA1* (primers ScMYBAS1_qPCR2_F and ScMYBAS1_qPCR2_R, Table 1). The ΔΔ^Ct^ method was used to analyse the expression level observed in the real-time PCR data [47]. To estimate the number of transgene copies, 24 rice transgenic lines were analysed by TaqMan-qPCR. Primers for the *hpt* II gene (hpt II_Taqman_F and hpt II_Taqman_R, Table 1) were designed from the pHb7m24GW vector sequence and for the endogenous *Sucrose Phosphate Synthase* (*SPS*) gene (SPS_Taqman_F and SPS_Taqman_R, Table 1), which has a single copy in the *O*. *sativa* genome [46]. They were used in the amplification reactions with the genomic DNA from the 12 *ScMYBAS1-2* rice transgenic lines and the 12 ScMYBAS1-3 rice transgenic lines plus the respective control plants (WT). The TaqMan qPCR amplification reactions were performed in quintuplicate in a multiplex reaction for the *hpt* II and *SPS* gene, containing 20 ng of DNA, 0.6 μM of each primer, 187.5 nM of each probe (*hpt* II probe and *SPS* probe, Table 1), 10 μL of TaqMan Fast Advanced Master Mix (Applied Biosystems) and water qsp for 20 μL. The copy number estimation of the transgene was done as described by [48]. Standard curves were prepared for the *hpt* II gene and the endogenous *SPS* gene. The Cycle Threshold (C_T_) values were used to obtain the starting quantities (SQ) for the hpt II and SPS genes. The r_line_ was obtained by the ratio between the SQ_hpt II_ and SQ_SPS_ for each rice transgenic line analysed. Based on the r_line_ values, the r_1_ coefficient (called ‘virtual calibrator’) was calculated for the *hpt* II gene using the data from all the transgenic and control plants. The copy number estimation of the transgene for each rice transgenic line was determined by the ratio between r_line_ and r_1_.

### Screening the T1 transgenic plants

The T_0_ transgenic rice lines, by natural self-pollination, produced T_1_ seeds in the greenhouse. The seeds from the T_1_ transgenic rice lines were sterilized for 5 min with 70% ethanol (v/v) and for 20 min with 5% NaClO (v/v) followed by five rinses with sterile distilled water. The sterile seeds (100 for each transgenic lines) were placed on MS3 agar medium supplemented with 50 mg L^-1^ hygromycin (Sigma, Santi Louis, MO, USA) and were maintained at 26°C, with a photoperiod of 14/10 h (light/dark) for 10 days [43]. The resistance to hygromycin was used to screen for the positive T_1_ transgenic plants. The performance of the surviving T_1_ seedlings was evaluated under the drought conditions.

### Drought treatment, plant water status and biometric analyses

Transgenic rice seedlings were transferred to polyvinyl chloride pots (5 L) containing a mixture (1:1, v/v) of soil and substrate (Carolina soil, São Paulo, SP, BR) and were grown in greenhouse conditions as follows: 25 ± 2/18 ± 2°C (day/night), 70 ± 5% relative humidity, 12/12 h (light/dark) photoperiod with natural light, 1100 μmol.m^−2^.s^−1^ average photosynthetic photon flux density (PPFD) (WatchDog Micro Station 1000 series, Spectrum Technologies, Aurora, IL, USA) across replications for daytime hours at Centro de Cana, Ribeirão Preto, São Paulo, Brazil (21.1704° S, 47.8103° W). Each pot contained one WT and one T_1_ transgenic rice seedlings, aiming to ensure that they were exposed to the same water availability [49]. When the plants were 30 days old, a water deficit was imposed by water withholding. A group of plants (control) was maintained in well-watered conditions through daily watering. After 10 days of a water deficit, the relative water content (RWC) was measured, using the leaf discs (1 cm^2^) collected at approximately 6 am [49]. For the biometric analysis, the wild-type and rice transgenic plants were harvested both at the beginning of the water deficit (one day after withholding) and at the maximum water deficit (10 days of water withholding), when plants presented a significant leaf wilting [50]. The soil water content was measured every two days during drought treatment, using a soil moisture sensor probe (ProCheck, Decagon Devices Inc, Pullman, WA, USA). The biometric traits, such as stalk length, root length, leaf length, tiller number and the dry weight of the root and shoots, were used for plant phenotyping.

## Results

### Sequences analysis of the ScMYBAS1 gene

**Fig 1:**
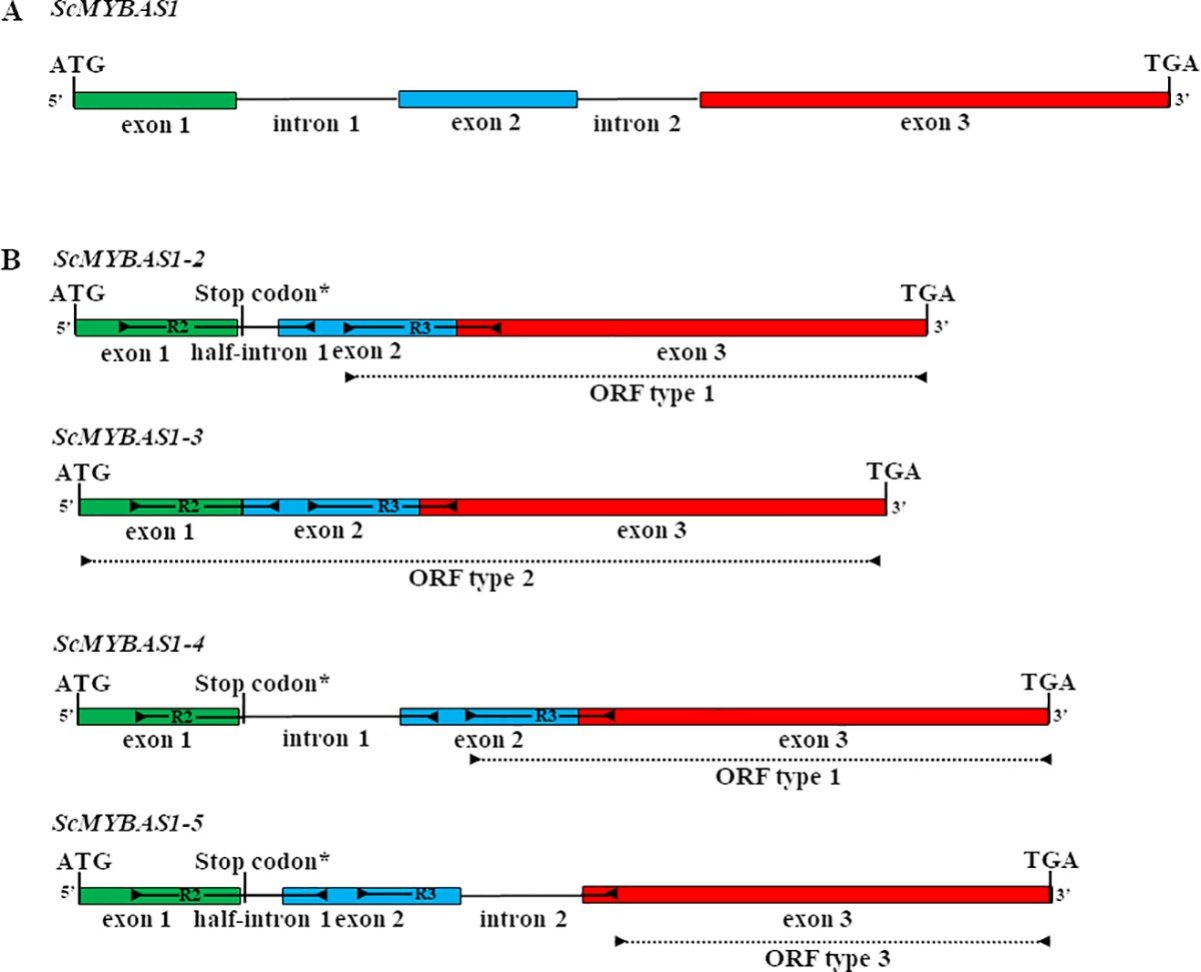
Schematic representation of the differently spliced transcripts of *ScMYBAS1*. (A) genomic DNA containing three exons (exon 1: 122 bp; exon 2: 131 bp and exon 3: 131 bp) and two introns (intron 1: 126 bp and intron 2: 86 bp. (B) differently spliced transcripts of *ScMYBAS1*, ScMYBAS1-2 (MH052202), ScMYBAS1-3 (MH052205), ScMYBAS1-4 (MH052203) and ScMYBAS1-5 (MH052201). The start codon ‘ATG’ and stop codon ‘TGA’ of each transcript are shown. The two MYB domain repeats (R2 and R3) and putative ORFs (Type 1, 2 and 3) are indicated in differently spliced transcripts.

Based on the SUCEST [34] and NCBI databases, the *ScMYBAS1* coding DNA sequence (CDS) was cloned from the cDNA of the sugarcane genotypes ‘IACSP94-2094’ and ‘IACSP97-7065’ via PCR. These genotypes were chosen, in this study, because they exhibit differential yield performance in drought-prone areas, with 'IACSP94-2094' being more tolerant to low water availability than 'IACSP97-7065' [51]. Forty-eight clones were sequenced in both directions (forward and reverse), and the assembled sequences resulted in 33 consensus sequences. The nucleotide sequence alignment (S1 Appendix) allowed us to classify the transcripts into four groups according to their size as follows: *ScMYBAS1-2* (716 bp); *ScMYBAS1-3* (687 bp); *ScMYBAS1-4* (813 bp) and *ScMYBAS1-5* (799 bp) (Fig 1).

**Fig 2:**
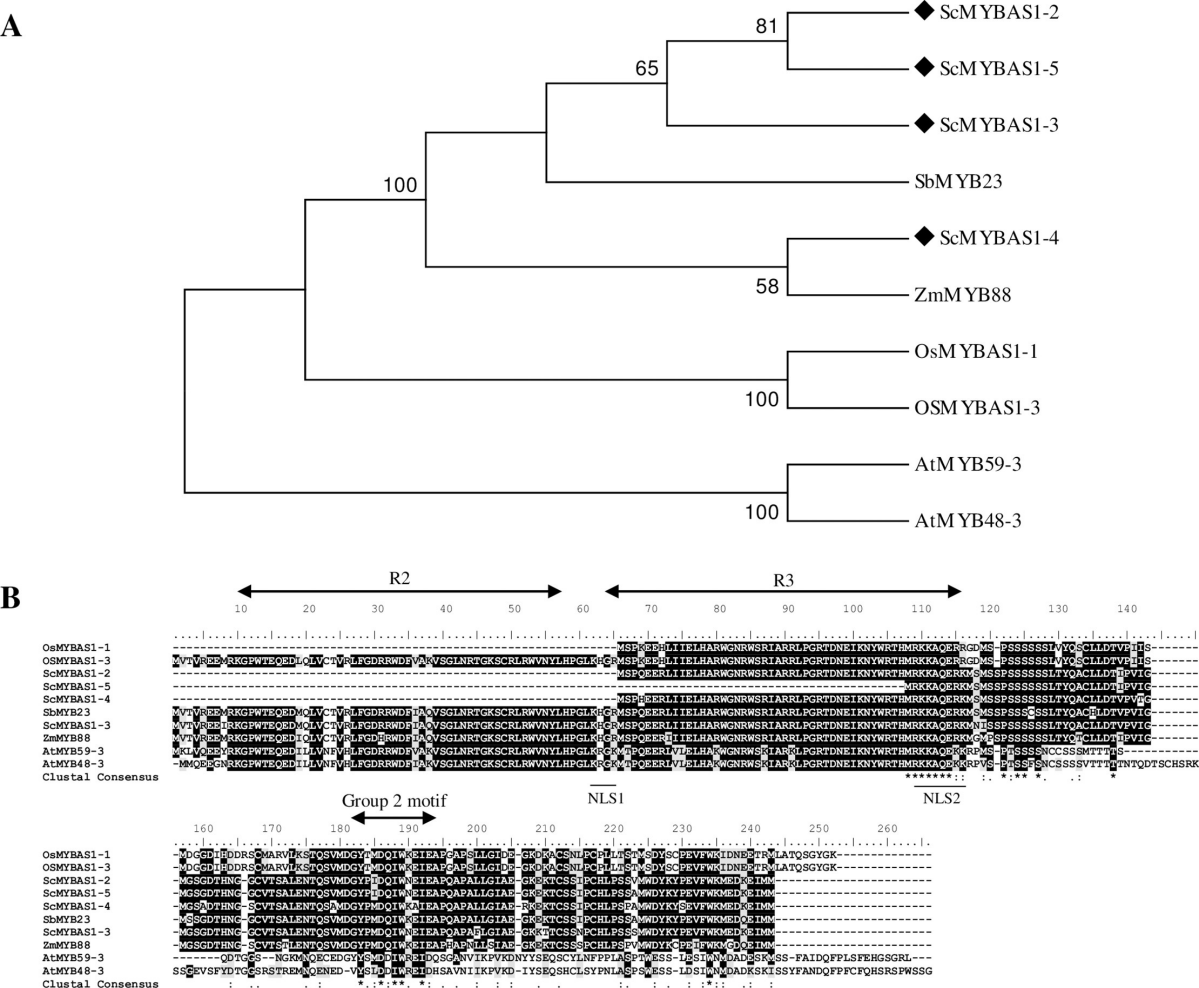
Dendrogram and sequence alignment of a subgroup of MYB protein sequences. (A) Dendrogram of a subgroup of MYB protein sequences from monocots and *A*. *thaliana*: ScMYBAS1-2 (MH052202), ScMYBAS1-3 (MH052205), ScMYBAS1-4 (MH052203), ScMYBAS1-5 (MH052201), SbMYB23 (Sobic.005G224800.1), ZmMYB88 (AFW60113.1), OsMYBAS1-1 (Q53NK6-2), OsMYBAS1-3 (AK111626), AtMYB59-3 (AT5G59780.3) and AtMYB48-3 (AT3G46130.1). Dendrogram was constructed with the parsimony method using MEGA 7, with a bootstrap of 100 samples. The branch numbers represent percentages of bootstrap. Sequence alignment of the proteins shown in (B). Identical amino acids are shaded in black, conserved changes in grey. The two MYB domain repeats (R2 and R3) and Group 2 motif are indicated with sets of arrows, and nuclear localization signals (NLS1 and NLS2) are indicated with black bars.

A dendrogram was built based on the translated amino acid sequences of ScMYBAS1-2, 3, 4, and 5 *Arabidopsis* members (AtMYB59-3 and AtMYB48-3) of a subgroup that was described as an alternative splicing/non-canonical intron subgroup [52] and the ScMYBAS1-2, 3, 4, and 5 first blast retrieved sequences of the monocots *Sorghum bicolor* (SbMYB23), *Zea mays* (ZmMYB88) and *Oryza sativa* (OsMYBSA1-1, OsMYBAS3–3) (Fig 2A). The monocot members clustered as more closely related than the Arabidopsis members. The ScMYBAS1-2, 3, 4, and 5 sequences showed an identity ranging from 64.2% to 95.1%, with other monocot members (S1 Table), and 53.5% and 58.1%, with the *Arabidopsis* AtMYB48-3 and AtMYB59-3 members, respectively. The c-terminus extremity of the ScMYBAS1-2, 3, 4, and 5 sequences had a YPMDQIWKEI conserved motif (Fig 2B), which is typical of Group 2 MYB family proteins classified according to Li, Li (53). The proteins encoded by *ScMYBAS1-2* and *ScMYBAS1-3* differed in only one amino acid at their C-terminus motif (I/M).

The transcript groups differed in the presence/combination of three intron sequences (intron 1: 126 bp, half-intron 1: 29 bp and intron 2: 88 bp), which were not removed during transcription splicing. Transcripts *ScMYBAS1-2* and *ScMYBAS1-4* showed an open read frame (ORF) type 1 that was responsible for encoding a putative protein with 163 amino acids (AAs) and a single incomplete R3 MYB repeat. Transcript *ScMYBAS1-3* had an ORF type 2, which encoded a protein with 228 AAs and two MYB repeats and was characteristic of an R2R3-MYB family gene. Transcript *ScMYBAS1-5* has an ORF type 3 (120 AAs) that showed no domain region related to MYB family. The longest ORF for each transcript was adopted as the putative ORF (Type 2). The most common transcript was *ScMYBAS1-2*, followed by *ScMYBAS1-5*, *ScMYBAS1-4* and *ScMYBAS1-3* at the ratio 26:4:2:1. The *ScMYBAS1-3* transcript was found only in the 'IACSP97-7065' genotype, and other transcripts were found in both genotypes. The transcripts ScMYBAS1-2 and ScMYBAS1-3 were chosen for the subsequent analyses. The transcripts ScMYBAS1-2 and ScMYBAS1-3 were chosen for the subsequent analyses. That because ScMYBAS1-2 showed higher transcripts abundance according to number of clones identified. In addition, it showed a premature stop codon, which encoded partial R2R2 MYB domain, similar to ScMYBAS1-4 and ScMYBAS1-5 transcripts (Fig 1), however, with lower clones abundance. Contrarily ScMYBAS1-3 showed the longest expected encoded protein (R2R3 MYB domain complete) and was identified only in the sensitive to drought genotype 'IACSP97-7065’.

### Subcellular localization

**Fig 3:**
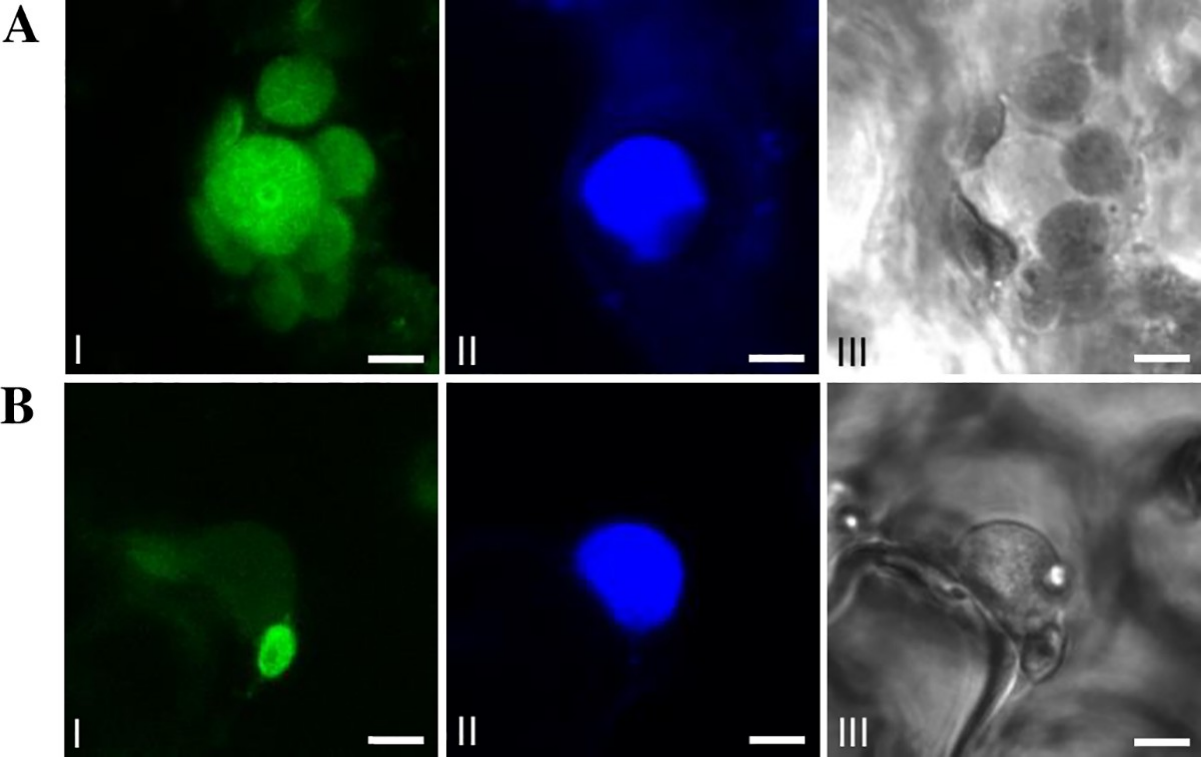
Subcellular localization of ScMYBAS1 proteins. The fusion constructs for ScMYBAS1-3:GFP (A) and ScMYBAS1-2:GFP (B) were introduced into *Nicotiana benthamiana* leaves via *Agrobacterium infiltration*. In (A) and (B) it is possible to verify a nuclear localization. Subcellular localization was investigated by confocal microscopy: I—GFP fluorescence; II—nucleus labeled with DAPI fluorescence; III—bright field image. Bars = 12.5 μm.

*Arabidopsis* R2R3-MYB proteins have highly conserved basic amino acid regions, namely, ‘KHGR’ and ‘RKKAQEKKM’, which serve as bipartite nuclear localization signals (NLS 1 and NLS2, respectively) [54]. NLS1 and NSL2 are present only in ScMYBAS1-3 transcript (Fig 2B), with one amino acid replacement in NLS2 region (‘RKKAQErKM’) whereas ScMYBAS1-2, ScMYBAS1-4 and ScMYBAS1-5 transcripts lacks the NLS1 region. To examine the subcellular localization of the proteins encoded by *ScMYBAS1-2* and *ScMYBAS1-3*, their coding sequences were cloned in frame to the 5´ N-terminus of the green fluorescent protein (GFP) reporter gene under the control of the cauliflower mosaic virus 35S promoter (CaMV 35S). Recombinant vectors (pScMYBAS1-2:GFP and pScMYBAS1-3:GFP) were introduced into *Nicotiana benthamiana* leaves via *Agrobacterium* infiltration. As shown in Fig 3, the ScMYBAS1-2:GFP and ScMYBAS1-3:GFP fusion proteins accumulated in the nucleus. Thus, they are both nuclear-localized proteins, which is consistent with their predicted function as a TF. ScMYBAS1-2:GFP was localized in the nucleus showing lower GFP fluorescence signal compared with ScMYBAS1-3:GFP (Fig 3B), problably due to the lack of NLS 1 in the ScMYBAS1-2. The MultiLoc online tool was used for an *in silico* subcellular localization. This tool shows the predicted targeting of proteins to different cell compartments, such as the nucleus, mitochondria, chloroplast, peroxisome and any other compartment [41]. The *in silico* analyses using the MultiLoc tool showed that ScMYBAS1-2 and ScMYBAS1-3 were possibly directed to the nucleus, with high scores of 0.61 and 0.86, respectively. Two other possibilities for the subcellular localization of ScMYBAS1-3 were extracellular and vacuolar, with scores of 0.05 and 0.02, respectively; and for ScMYBAS1-2, the possibilities were the peroxisome, cytoplasm and secretory pathway, with scores of 0.27, 0.21 and 0.18, respectively.

### Transgenic plants under a water deficit

**Fig 4:**
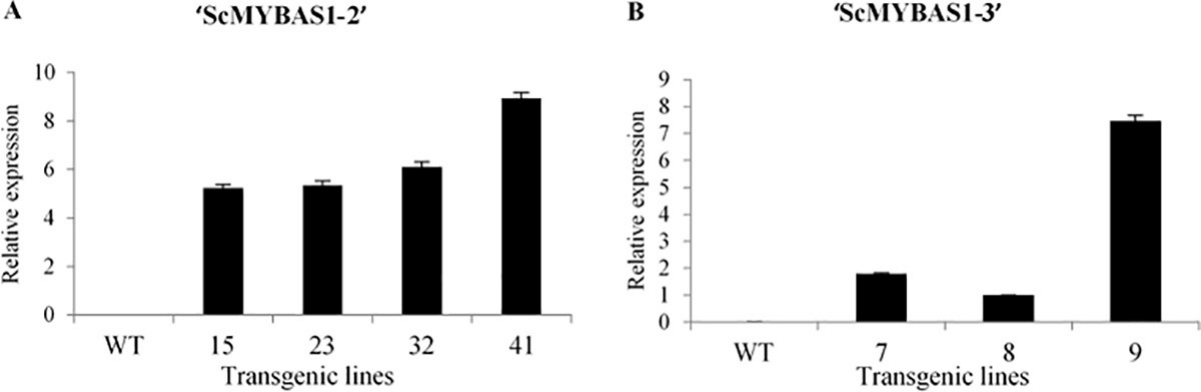
Relative expression of *ScMYBAS1* gene in transgenic rice lines and wild type (WT) under greenhouse conditions. The leaves of 3-week-old seedling weres sampled for RNA extraction. Relative expression levels were obtained from the ratio of transgenic line with the lowest expression level and the other transgenic lines. *Elongation factor 1 alpha* (*eEF1a*) was used as a housekeeping gene. A, transgenic ScMYBAS1-2 lines; and B transgenic ScMYBAS1-3 lines used in physiological and biometric analyses. The values are mean ± SE (n = 3).

Rice calli were transformed with two different overexpression vectors carrying the *ScMYBAS1-2* and *ScMYBAS1-3* coding sequences under the control of the maize *Ubiquitin* promoter. The transformation showed a high efficiency, i.e., 50–60%, as given by the ratio of the transgenic plants produced to the number of the inoculated calli with *Agrobacterium*. Several rice transgenic plants overexpressing *ScMYBAS1-2* (30 independent events of plants) and *ScMYBAS1-3* (25 independent events of plants) were obtained, and all the events were selected by hygromycin resistance and confirmed by PCR. Four *ScMYBAS1-2* transgenic lines (identified as 15, 23, 32, and 41) and three *ScMYBAS1-3* transgenic lines (identified as 7, 8 and 9) from the first segregate generation, containing a single copy of the transgene (Table 2) and showing different expression levels (Fig 4), were subjected to a water deficit treatment.

**Table 2:** Taqman qPCR estimates of copy number for *hpt* II transgene of ScMYBAS1-2 and ScMYBAS1-3 transgenic plants.

Transgenic Lines		r_*line*_	r_*line*_/r_*1*_	Copy Number *hpt* II
**‘ScMYBAS1-2’**	**1**	2.57 ± 0.156	4.28	4
	**3**	0.60 ± 0.008	1.00	1
	**7**	6.40 ± 0.253	10.67	11
	**8**	8.22 ± 0.328	13.69	14
	**15**	0.57 ± 0.026	0.94	1
	**16**	1.30 ± 0.021	2.16	2
	**20**	0.78 ± 0.014	1.31	1
	**23**	0.55 ± 0.015	0.91	1
	**32**	0.59 ± 0.010	0.98	1
	**34**	0.42 ± 0.009	0.71	1
	**38**	0.16 ± 0.008	0.26	1*
	**41**	0.79 ± 0.023	1.32	1
**‘ScMYBAS1-3’**	**4**	1.40 ± 0.035	1.75	2
	**5**	1.22 ± 0.082	1.52	2
	**7**	0.12 ± 0.028	0.15	1*
	**8**	0.17 ± 0.019	0.21	1*
	**9**	0.17 ± 0.036	0.21	1*
	**11**	0.32 ± 0.036	0.40	1*
	**21**	1.51 ± 0.067	1.88	2
	**25**	1.85 ± 0.065	2.31	2
	**26**	0.46 ± 0.070	0.58	1
	**28**	0.78 ± 0.035	0.98	1
	**31**	2.15 ± 0.054	2.69	3
	**32**	2.42 ± 0.093	3.08	3

*Values assumed as a single copy.

**Fig 5:**
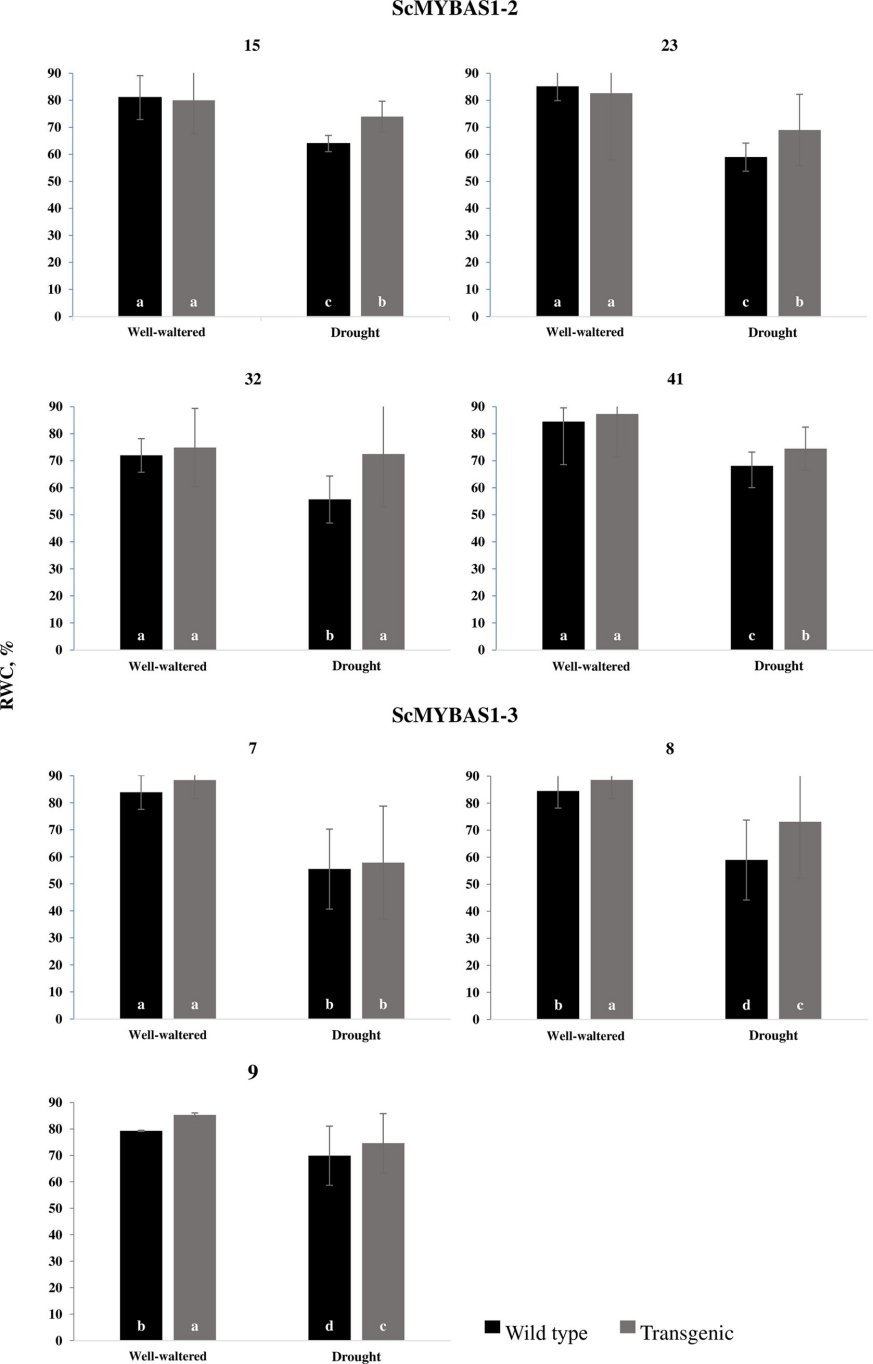
Leaf relative water content (RWC) in wild type and transgenic lines under well-watered and drought stress condition. The values are mean ± SE (n = 4). The letters on the bars indicate significant differences by Student’s t-test (P < 0.05).

The *ScMYBAS1-2* and *ScMYBAS1-3* transgenic lines were tested at 30 days of age under varying water conditions. After 10 days of water withholding, transgenic and WT plants presented a significant leaf wilting and a significant reduction of soil water content (S1 Fig) characterizing the drought treatment. The RWC of the transgenic lines and controls (WT) was measured after drought treatment (Fig 5). Before stress, the S*cMYBAS1-2* transgenic lines and WT did not show a significant difference in the leaf RWC, which ranging from 72% to 88%. Under drought conditions, all the *ScMYBAS1-2* lines exhibited a higher RWC than did the WT (Fig 5). The *ScMYBAS1-3* transgenic lines 8 and 9 showed a higher RWC than did the WT before and after water stress, while line 7 and the WT had a similar RWC (Fig 5).

**Fig 6:**
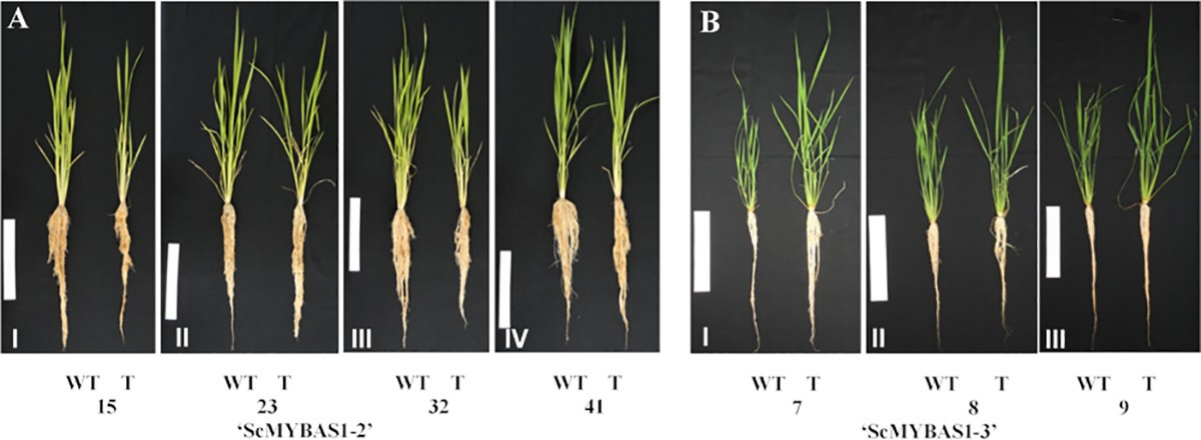
Phenotypes of T_1_ transgenic rice overexpressing *ScMYBAS1-2* and *ScMYBAS1-3*. A: ScMYBAS1-2 transgenic lines after 45 days growth in greenhouse conditions, I—IV: 15, 23, 32 and 41. B: ScMYBAS1-3 transgenic lines after 30 days growth in greenhouse conditions, V—VII: 7, 8 and 9. Left: WT plants; Right: transgenic plants. White bar = 20 cm.

The *ScMYBAS1-2* transgenic lines showed a reduction in the biomass in the well-watered and drought treatments (Table 3). The biomass reduction (total dry weight) of the transgenic lines was more evident in the well-watered group than in the drought treatment group. Conversely, the transgenic lines 7, 8 and 9 of *ScMYBAS1-3* showed an increased biomass (total dry weight) relative to the WT (Table 4), and this increase was higher in the drought treatment (Fig 6).

**Table 3:** Biometric parameters evaluated in the transgenic lines ScMYBAS1-2 under well-watered and drought conditions at maximum stress.

Variables	15				23				32				41			
	Well-watered		Drought		Well-watered		Drought		Well-watered		Drought		Well-watered		Drought	
	WT	T	WT	T	WT	T	WT	T	WT	T	WT	T	WT	T	WT	T
**Tillers (units)**	6.0±1.4a	3.3±0.8b	7.0±0.8a	4.0±0.1b	5.7±1.7a	4.0±0.0c	5.2±1.2ab	4.5±0.6bc	6.2±1.2a	3.0±0.0c	5.2±0.5b	4.5±0.6b	4.7±0.5a	4.2±0.5a	4.7±0.5a	4.5±1.7a
**Leaf length (cm)**	19.4±3.3b	15.0±3.7c	22.2±4.2a	24.4±6.4a	22.1±5.7b	24.5±2.8a	22.6±5.3b	26.7±7.0a	20.6±2.6b	21.6±3.1ab	24.1±1.5ab	25.0±6.2a	24.4±5.0ab	19.8±2.9b	28.0±1.0a	24.9±2.7ab
**Stalk length (cm)**	38.6±0.7a	28.5±2.3c	34.5±3.1b	26.3±4.0c	37.1±5.4a	34.6±0.7a	34.3±0.4a	29.0±2.2b	37.4±1.9a	29.5±3.0b	35.4±0.4a	29.5±3.1b	35.6±2.9a	30.5±2.4b	34.8±3.0a	30.6±1.6b
**Root length (cm)**	42.8±0.6	43.8±9.4	39.9±7.6a	36.5±6.6a	39.2±4.8a	35.9±3.5b	38.6±2.0a	41.0±3.0a	42.4±4.6ab	45.6±3.8a	37.7±4.4b	37.4±3.2b	38.9±2.2a	38.4±5.5a	38.6±3.2a	34.5±4.3b
**Root dry matter (g)**	1.5±0.2a	0.6±0.1b	1.5±0.3a	0.7±0.1b	1.6±0.3a	1.0±0.2b	1.2±0.2a	1.0±0.4b	1.7±0.1a	0.8±0.1c	1.3±0.1b	1.3±0.5b	1.6±0.4a	1.0±0.2c	1.3±0.1b	0.9±0.2c
**Shoot dry matter (g)**	4.5±0.8b	1.1±0.3d	5.0±0.9a	1.9±0.3c	4.8±1.7a	2.8±0.2b	4.4±0.8a	3.2±0.8b	4.7±0.6a	1.2±0.1c	4.8±0.5a	3.4±1.0b	4.3±1.3a	2.0±1.1c	4.4±0.4a	3.1±0.4b
**Dry matter (g)**	6.0±0.9a	1.7±0.3b	6.6±1.1a	2.6±0.3b	6.3±2.0a	3.8±0.3b	5.7±1.0a	4.2±1.1b	6.4±0.7a	2.0±0.2c	6.5±1.2a	3.7±1.9b	5.9±1.7a	3.0±0.2b	5.7±0.4a	3.9±0.6b

The values are mean ± SE (n = 4). The letters on the same lines indicate significant difference by Student’s t-test (P < 0.05) within the same event.

**Table 4:** Biometric parameters evaluated in the transgenic lines ScMYBAS1-3 under well-watered and drought conditions at maximum stress.

Variables	7				8				9			
	Well-watered		Drought		Well-watered		Drought		Well-watered		Drought	
	WT	T	WT	T	WT	T	WT	T	WT	T	WT	T
**Tillers (units)**	10.0±0.8b	13.2±2.4a	8.7±1.9b	11.5±2.4ab	10.2±1.2ab	11.5±1.9a	8.7±1.2b	8.5±2.4b	11.2±1.7b	15.0±3.5a	8.2±2.0c	10.0±1.4bc
**Leaf length (cm)**	22.0±2.6bc	28.0±1.8a	17.0±4.2c	25.0±6.2ab	20.7±4.5b	30.8±3.3a	16.6±3.8b	31.9±9.4a	22.2±3.0b	32.5±1.2a	19.4±3.0b	28.6±7.3a
**Stalk length (cm)**	23.5±2.9b	30.6±0.3a	22.7±2.4b	28.2±1.7a	29.3±2.0	30.3±5.4	20.5±4.8b	23.4±2.9a	24.5±3.0ab	28.7±4.0a	21.8±2.4b	25.8±6.2ab
**Root length (cm)**	39.3±2.4a	38.4±0.7a	42.2±5.6a	42.9±7.2a	48.0±0.8a	40.8±2.7b	34.7±2.9c	41.6±4.5b	38.0±5.3a	41.7±6.1a	39.3±4.6a	39.9±5.4a
**Root dry matter (g)**	0.5±0.1b	0.7±0.1a	0.4±0.1c	0.6±0.05b	0.8±0.02a	0.9±0.2a	0.4±0.1a	0.6±0.4a	0.5±0.1bc	0.9±0.3a	0.4±0.04cd	0.6±0.2b
**Shoot dry matter (g)**	1.8±0.4b	2.8±0.1a	1.0±0.2c	1.8±0.1b	2.7±0.1b	3.4±0.6a	1.2±0.2d	1.9±1.2c	1.9±0.6b	4.1±1.3a	1.1±0.2c	1.8±0.7b
**Dry matter (g)**	2.4±0.5b	3.5±0.1a	1.4±0.3c	2.4±0.2b	3.5±0.1a	4.2±0.8a	1.7±0.3b	2.5±1.6b	2.5±0.7b	5.0±1.6a	2.5±0.7b	5.0±1.6a

The values are mean ± SE (n = 4). The letters on the same lines indicate significant difference by Student’s t-test (P < 0.05) within the same event.

## Discussion

In the present study, we identified four groups of *ScMYBAS1* transcripts, including *ScMYBAS-2*, *ScMYBAS-3*, *ScMYBAS-4* and *ScMYBAS-5*, which were putative splicing alternatives because they shared a similar alternative splicing pattern to Arabidopsis and rice transcription factors (*AtMYB48*, *AtMYB59*, *OsMYBAS1* and *OsMYBAS2*) (Li et al. 2006a). In sugarcane, Guo, Ling (31) described a highly conserved splicing arrangement with three exons and two introns for *ScMYBAS1* (named as *ScMYB2*). The introns found by Guo, Ling (31) and in our study have specific splice sites (GT-AG) [55]. Based on the results herein, it is possible that *ScMYBAS1-4* and *ScMYBAS1-5* also undergo the same conserved alternative splicing. *ScMYBAS1* encodes an MYB protein with one or two MYB repeats (R2 and R3), which are known to bind to DNA. The proteins of the transcripts *ScMYBAS-2*, *ScMYBAS-3* and *ScMYBAS-4*, encoded by *ScMYBAS1*, differ in their MYB domain repeats. Therefore, these three types of MYB proteins may have binding affinities to different target genes. The alternative splicing of transcripts is one of the most complex cellular processes in eukaryotes that leads to a large proportion of proteomic complexity without genome expansion [56]. Alternative splicing is documented for mRNAs originating from several plant transcription factor genes and is frequently associated with environmental stress [57, 58]. ScMYABS1 alternative spliced transcripts act differently in regulating the pathways involved in drought tolerance. Once exposed to drought conditions, the genotypes 'IACSP 97–7065' and 'IACSP94-2094', with a response profile that contrasts to drought stress, have different frequencies and types of ScMYBAS1 transcripts. The *ScMYBAS1-3* transcript with an ORF type 2, encoding the longest protein (228 AAs), was identified only in the genotype 'IACSP97-7065' and was considered sensitive to drought, whereas the *ScMYBAS1-5* variant with an ORF type 3 was observed only in the genotype 'IACSP94-2094' and was considered tolerant to drought conditions. Conversely, the most common *ScMYBAS1-2* and *ScMYBAS1-4* transcripts with an ORF type 1 (Fig 1B) were found in both sugarcane genotypes studied here. To our knowledge, *ScMYBAS1-4* and *ScMYBAS1-5* were identified for the first time herein, whereas *ScMYBAS1-2* and *ScMYBAS1-3* were first identified by Prabu and Theertha [30] in the sugarcane seedlings of the Co740 genotype under water-deficit and salt stress conditions. Recently, these same transcripts were identified in sugarcane exposed to drought and were named *ScMYB2S1* and *ScMYB2S2* [31]. According to the authors, *ScMYB2S1* and *ScMYB2S* are involved in the ABA-mediated leaf senescence signalling pathway and play a positive role in response to drought-induced senescence in sugarcane.

The distribution of conserved AAs among the MYB DNA-binding domain (DBD) of sugarcane, sorghum, maize, and rice (phylogenetically related plants), including *Arabidopsis*, was very similar, indicating that the AA residues in this domain were highly conserved across plants (Fig 2). The amino acid residues of the DBD (R2 and R3 domains) are important functional domains of the MYB family. Despite the high similarity in the DBD (Fig 2), it was possible to cluster the MYB proteins according to the phylogenetic relationships between monocots and dicots. The dendrogram was composed of all translated AA sequences from the *ScMYBAS1* transcripts and the sequences of sorghum (*SbMYB23*), maize (*ZmMYB88*) and rice (*OsMYBAS1*), revealing a higher similarity among monocot than dicot proteins. The *Arabidopsis* protein sequences probably represent orthologues to ScMYBAS1 [31]. The clustering was possible due the variability in the C-terminal region of the MYB proteins. Moreover, most plant MYB proteins are composed of a set of conserved motifs in the C-terminal, and the protein architectures are remarkably conserved within specific MYB subgroups. Members of the same MYB subgroups generally share one or more identical motifs outside the MYB DBD [8, 12, 59]. In general, the C-terminal regions of the MYB proteins often possess a protein-protein interaction domain. *ScMYBAS1* contains the motif YPMDQIWKEI, in the C-terminus of the protein, and this is similar to motif 52 (W/Y-MDDIW) in *Arabidopsis*, which is reported to be a transcriptional activator [59]. This motif is a conserved sequence in the C-termini among the proteins, suggesting its functional importance.

It is well known that MYB proteins play important roles in multiple aspects of regulating responses to abiotic stress, plant growth and development [12, 13, 60–62]. To examine the response to drought stress in the transgenic lines, the T_1_ progenies of the *ScMYBAS1-2* and *ScMYBAS1-3* transgenic lines and WT plants were grown in a greenhouse under well-watered and water-withholding conditions. The *ScMYBAS1-2* and *ScMYBAS1-3* transgenic lines exhibited significant physiological differences in their RWC parameters compared to the WT plants. Surprisingly, the *ScMYBAS1-3* transgenic lines showed an increased biomass, whereas the *ScMYBAS1-2* transgenic lines revealed a decreased biomass under both the well-watering and drought stressed conditions, and there was a higher RWC in the transgenic lines compared with the WT. Although there was a reduction in the biomass in *ScMYBAS1-2* transgenic lines in both water regimes, this reduction was lower in the drought-stressed treatment. This fact may be related to the high RWC in the transgenic lines exposed to drought. In *ScMYBAS1-3* transgenic lines, the increase in the biomass was higher in the drought conditions. As in the *ScMYBAS1-2* transgenic lines, the RWC in the *ScMYBAS1-3* transgenic lines was higher than that of the WT. The RWC is a measure of the plant water status that reflects the metabolic activity in tissues, and it is used as the most meaningful index of water stress tolerance [63]. A decrease in the RWC in response to drought stress is noted in a wide variety of plants. Leaves subjected to drought exhibit large reductions in the RWC and water potential [62]. Transgenic plants that present a delay in water loss compared to non-transgenic plants are characterized as resistant to drought [64]. A delay in water loss during drought allows transgenic plants to maintain their homeostasis for a longer time than the WT plants, conferring an advantage over stress. This event was observed in the ScMYBAS1-2 transgenic lines 15, 23, 32 and 41 and the ScMYBAS1-3 transgenic lines 8 and 9, which presented a higher RWC than did the WT plants under drought conditions, thus characterizing the involvement of *ScMYAS1-2* and *ScMYBAS1-3* in the drought tolerance. However, from the agronomic point of view, a reduction in the biomass in the ScMYAS1-2 transgenic lines is a negative aspect for sugarcane cultivation. On the other hand, the ScMYBAS1-3 transgenic lines 8 and 9 exhibited an increase in biomass (more evident in the line 9, which coincides with the higher transgene expression level compared to the three transgenic lines evaluated) and a larger RWC in relation to the WT plants. The high relative RWC found in the transgenic lines suggests that *ScMYAS1-3* plays a positive role in response to drought.

*ScMYBAS1* was found to undergo alternative splicing and produced four distinctively spliced transcripts. Interestingly, the encoded proteins for *ScMYBAS1-2* and *ScMYBAS1-3* mainly differ in their MYB DBD repeat and in one amino acid in the C-terminus motif (I/W). Similarly, the *ScMYBAS1* orthologues in *Arabidopsis*, *AtMYB59* and *AtMYB48*, were found to undergo highly conserved alternative splicing [59], and the proteins encoded by the *AtMYB59-1* and *AtMYB59-3* transcripts were transcriptional activators, suggesting that these transcription factors may activate different groups of target genes by changing their DNA binding domains *via* alternative splicing [53]. *ScMYBAS1-2* and *ScMYBAS1-3* possibly act in a manner similar to their *AtMYB59* and *AtMYB48* orthologues by activating different groups of target genes, resulting in a high RWC and contrasting biomass in transgenic rice. The transcription factor *AtMYB59* is an important regulator of the cell cycle [65]. The transgenic plants of *Arabidopsis* overexpressing this TF showed a reduction in root size relative to the wild-type plants. In contrast, mutant *AtMYB59* lines showed an increase in root length also in relation to the wild-type plants. The transgenic lines overexpressing *AtMYB59* presented 54% of the cells in metaphase, whereas the wild-type and mutant strains presented only 27% and 14% of the cells, respectively, in this phase. This extension in the metaphase phase during cell division resulted in slower root growth in the transgenic lines, thereby impairing their normal development. Further studies are needed to identify the possible targets of these distinctively spliced transcripts and the correlation between the MYB domain and the trans-activation activity.

Since drought tolerance and an increase in biomass are highly desirable traits in energy crops, the overexpression of *ScMYBAS1-3* may be an excellent target to be explored in the generation of transgenic plants, such as sugarcane, energy cane and other bioenergy grasses. Indeed, the manipulation of only one gene contributes to an increase in the productivity per area, promoting the verticalization of crop production, inside a sustainable production context. Moreover, with the advent of technologies based on genome editing, *ScMYBAS1-3* is considered suitable for precise genome editing, bypassing the biosafety and regulatory aspects that lie with genetically modified plants.

## Supporting information

S1 Appendix Sequence alignment of the *ScMYBAS1* nucleotide sequence showing different transcripts from ‘IACSP94-2094’ and ‘IACSP97-7065’ sugarcane cultivars. (PDF)

S1 Fig Variation of soil moisture (θ, m^3^ m^-3^) measured every two days during well-watering and water withholding treatments. The values are mean ± SE (n = 4).
(TIF)

S1 Table Percent identity matrix of a subgroup of MYB protein sequences. Protein sequences from monocots and *A*. *thaliana*: ScMYBAS1-2 (MH052202), ScMYBAS1-3 (MH052205), ScMYBAS1-4 (MH052203), ScMYBAS1-5 (MH052201), SbMYB23 (Sobic.005G224800.1), ZmMYB88 (AFW60113.1), OsMYBAS1-1 (Q53NK6-2), OsMYBAS1-3 (AK111626), AtMYB59-3 (AT5G59780.3) and AtMYB48-3 (AT3G46130.1).
(PDF)
