# Supplementary material for: Overexpression of ScMYBAS1 alternative splicing transcripts differentially impacts biomass accumulation and drought tolerance in rice transgenic plants
Source: PLoS One. 2018 Dec 5;13(12):e0207534. doi: 10.1371/journal.pone.0207534 (PMC6281192; doi:10.1371/journal.pone.0207534)
Supplement: S1 Appendix — (PDF) [file pone.0207534.s001.pdf]

S1 Appendix. Sequence alignment of the ScMYBAS1 nucleotide sequence showing different transcripts from ‘IACSP94-2094’ and ‘IACSP97-7065’ sugarcane cultivars.

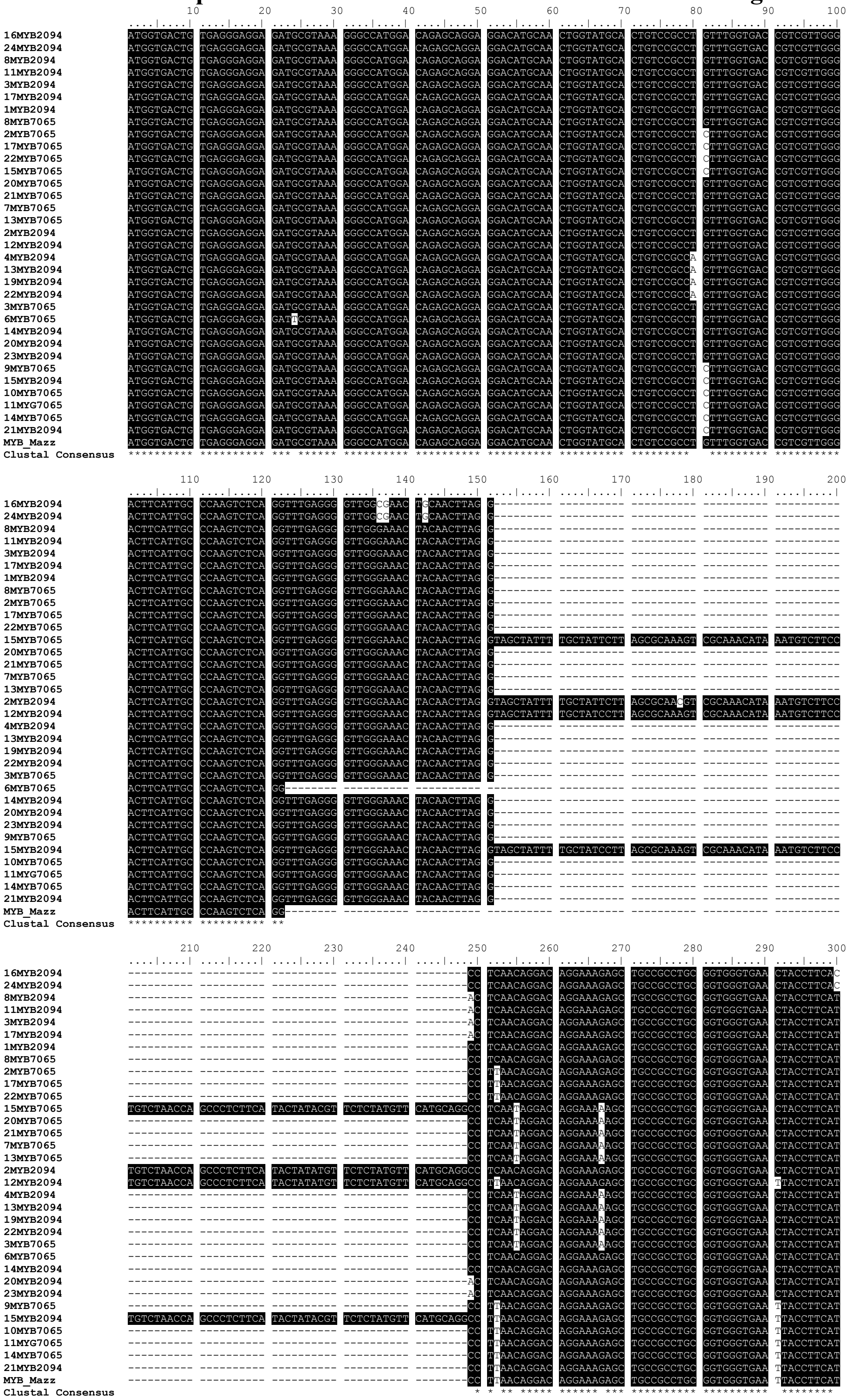

```
                           310        320        330        340        350        360        370        380        390        400
                   ....|....| ....|....| ....|....| ....|....| ....|....| ....|....| ....|....| ....|....| ....|....| ....|....|
16MYB2094          CCTGGCCTCA AGCATGGCAG CATGTCACCA CAAGAAGAAC GCCTTATTAT TGAGCTCCAT GCTCGGTGGG GGAACAGGT- ---------- ----------
24MYB2094          CCTGGCCTCA AGCATGGCAG CATGTCACCA CAAGAAGAAC GCCTTATTAT TGAGCTCCAT GCTCGGTGGG GGAACAGGT- ---------- ----------
8MYB2094           CCTGGCCTCA AGCATGGCCG CATGTCACCA CAAGAAGAAC GCCTTATTAT TGAGCTGCAT GCTCGGTGGG GGAACAGGTT TGATCTGAAG TTTTTCTAGA
11MYB2094          CCTGGCCTCA AGCATGGCCG CATGTCACCA CAAGAAGAAC GCCTTATTAT TGAGCTGCAT GCTCGGTGGG GGAACAGGTT TGATCTGAAG TTTTTCTAGA
3MYB2094           CCTGGCCTCA AGCATGGCCG CATGTCACCA CAAGAAGAAC GCCTTATTAT TGAGCTGCAT GCTCGGTGGG GGAACAGGT- ---------- ----------
17MYB2094          CCTGGCCTCA AGCATGGCCG CATGTCACCA CAAGAAGAAC GCCTTATTAT TGAGCTGCAT GCTCGGTGGG GGAACAGGT- ---------- ----------
1MYB2094           CCTGGCCTCA AGCATGGCCG CATGTCACCA CAAGAAGAAC GCCTTATTAT TGAGCTGCAT GCTCGGTGGG GGAACAGGT- ---------- ----------
8MYB7065           CCTGGCCTCA AGCATGGCCG CATGTCACCA CAAGAAGAAC GCCTTATTAT TGAGCTCCAT GCTCGGTGGG GGAACAGGT- ---------- ----------
2MYB7065           CCTGGCCTCA AGCATGGCCG CATGTCACCA CAAGAAGAAC GCCTTATTAT TGAGCTCCAT GCTCGGTGGG GGAACAGGT- ---------- ----------
17MYB7065          CCTGGCCTCA AGCATGGCCG CATGTCACCA CAAGAAGAAC GCCTTATTAT TGAGCTCCAT GCTCGGTGGG GGAACAGGT- ---------- ----------
22MYB7065          CCTGGCCTCA AGCATGGCCG CATGTCACCA CAAGAAGAAC GCCTTATTAT TGAGCTCCAT GCTCGGTGGG GGAACAGGT- ---------- ----------
15MYB7065          CCTGGCCTCA AGCATGGCCG CATGTCACCA CAAGAAGAAC GCCTTATTAT TGAGCTCCAT GCTCGGTGGG GGAACAGGT- ---------- ----------
20MYB7065          CCTGGCCTCA AGCATGGCCG CATGTCACCA CAAGAAGAAC GCCTTATTAT TGAGCTCCAT GCTCGGTGGG GGAACAGGT- ---------- ----------
21MYB7065          CCTGGCCTCA AGCATGGCCG CATGTCACCA CAAGAAGAAC GCCTTATTAT TGAGCTCCAT GCTCGGTGGG GGAACAGGT- ---------- ----------
7MYB7065           CCTGGCCTCA AGCATGGCCG CATGTCACCA CAAGAAGAAC GCCTTATTAT TGAGCTCCAT GCTCGGTGGG GGAACAGGT- ---------- ----------
13MYB7065          CCTGGCCTCA AGCATGGCCG CATGTCACCA CAAGAAGAAC GCCTTATTAT TGAGCTCCAT GCTCGGTGGG GGAACAGGT- ---------- ----------
2MYB2094           CCTGGCCTCA AGCATGGCCG CATGTCACCA CAAGAAGAAC GCCTTATTAT TGAGCTCCAT GCTCGGTGGG GGAACAGGT- ---------- ----------
12MYB2094          CCTGGCCTGA AGCATGGCCG CATGTCACCA CACGAAGAAC GCCTTATTAT TGAGCTCCAT GCTCGGTGGG GGAACAGGT- ---------- ----------
4MYB2094           CCTGGCCTCA AGCATGGCCG CATGTCACCA CAAGAAGAAC GCCTTATTAT TGAGCTGCAT GCTCGGTGGG GGAACAGGT- ---------- ----------
13MYB2094          CCTGGCCTCA AGCATGGCCG CATGTCACCA CAAGAAGAAC GCCTTATTAT TGAGCTGCAT GCTCGGTGGG GGAACAGGT- ---------- ----------
19MYB2094          CCTGGCCTCA AGCATGGCCG CATGTCACCA CAAGAAGAAC GCCTTATTAT TGAGCTGCAT GCTCGGTGGG GGAACAGGT- ---------- ----------
22MYB2094          CCTGGCCTCA AGCATGGCCG CATGTCACCA CAAGAAGAAC GCCTTATTAT TGAGCTGCAT GCTCGGTGGG GGAACAGGT- ---------- ----------
3MYB7065           CCTGGCCTCA AGCATGGCCG CATGTCACCA CAAGAAGAAC GCCTTATTAT TGAGCTCCAT GCTCGGTGGG GGAACAGGT- ---------- ----------
6MYB7065           CCTGGCCTCA AGCATGGCCG CATGTCACCA CAAGAAGAAC GCCTTATTAT TGAGCTCCAT GCTCGGTGGG GGAACAGGT- ---------- ----------
14MYB2094          CCTGGCCTCA AGCATGGCCG CATGTCACCA CAAGAAGAAC GCCTTATTAT TGAGCTCCAT GCTCGGTGGG GGAACAGGT- ---------- ----------
20MYB2094          CCTGGCCTCA AGCATGGCCG CATGTCACCA CAAGAAGAAC GCCTTATTAT TGAGCTGCAT GCTCGGTGGG GGAACAGGT- ---------- ----------
23MYB2094          CCTGGCCTCA AGCATGGCCG CATGTCACCA CAAGAAGAAC GCCTTATTAT TGAGCTGCAT GCTCGGTGGG GGAACAGGT- ---------- ----------
9MYB7065           CCTGGCCTGA AGCATGGCCG CATGTCACCA CACGAAGAAC GCCTTATTAT TGAGCTCCAT GCTCGGTGGG GGAACAGGT- ---------- ----------
15MYB2094          CCTGGCCTGA AGCATGGCCG CATGTCACCA CACGAAGAAC GCCTTATTAT TGAGCTCCAT GCTCGGTGGG GGAACAGGT- ---------- ----------
10MYB7065          CCTGGCCTGA AGCATGGCCG CATGTCACCA CACGAAGAAC GCCTTATTAT TGAGCTCCAT GCTCGGTGGG GGAACAGGT- ---------- ----------
11MYG7065          CCTGGCCTGA AGCATGGCCG CATGTCACCA CACGAAGAAC GCCTTATTAT TGAGCTCCAT GCTCGGTGGG GGAACAGGT- ---------- ----------
14MYB7065          CCTGGCCTGA AGCATGGCCG CATGTCACCA CACGAAGAAC GCCTTATTAT TGAGCTCCAT GCTCGGTGGG GGAACAGGT- ---------- ----------
21MYB2094          CCTGGCCTGA AGCATGGCCG CATGTCACCA CACGAAGAAC GCCTTATTAT TGAGCTCCAT GCTCGGTGGG GGAACAGGT- ---------- ----------
MYB_Mazz           CCTGGCCTGA AGCATGGCCG CATGTCACCA CACGAAGAAC GCCTTATTAT TGAGCTCCAT GCTCGGTGGG GGAACAGGT- ---------- ----------
Clustal Consensus  ******** * ******** * ********** ** ******* ********** ****** *** ********** *********

                           410        420        430        440        450        460        470        480        490        500
                   ....|....| ....|....| ....|....| ....|....| ....|....| ....|....| ....|....| ....|....| ....|....| ....|....|
16MYB2094          ---------- ---------- ---------- ---------- ---------- ---------- -------GGT CTAGGATAGC ACGGAGGCTG CCAGGGCGTA
24MYB2094          ---------- ---------- ---------- ---------- ---------- ---------- -------GGT CTAGGATAGC ACGGAGGCTG CCAGGGCGTA
8MYB2094           GAGTAACTTT TCTCAGTTAC TAAATTTAGG TGAAGACAAA GCTCACTCAA ATTCTCCATA TGTAGGTGGT CTAGGATAGC ACGGAGGCTG CCAGGGCGTA
11MYB2094          GAGTAACTTT TCTCAGTTAC TAAATTTAGG TGAAGACAAA GCTCACTCAA ATTCTCCATA TGTAGGTGGT CTAGGATAGC ACGGAGGCTG CCAGGGCGTA
3MYB2094           ---------- ---------- ---------- ---------- ---------- ---------- -------GGT CTAGGATAGC ACGGAGGCTG CCAGGGCGTA
17MYB2094          ---------- ---------- ---------- ---------- ---------- ---------- -------GGT CTAGGATAGC ACGGAGGCTG CCAGGGCGTA
1MYB2094           ---------- ---------- ---------- ---------- ---------- ---------- -------GGT CTAGGATAGC ACGGAGGCTG CCAGGGCGTA
8MYB7065           ---------- ---------- ---------- ---------- ---------- ---------- -------GGT CTAGGATAGC ACGGAGGCTG CCAGGGCGTA
2MYB7065           ---------- ---------- ---------- ---------- ---------- ---------- -------GGT CTAGGATAGC ACGGAGGCTG CCAGGGCGTA
17MYB7065          ---------- ---------- ---------- ---------- ---------- ---------- -------GGT CTAGGATAGC ACGGAGGCTG CCAGGGCGTA
22MYB7065          ---------- ---------- ---------- ---------- ---------- ---------- -------GGT CTAGGATAGC ACGGAGGCTG CCAGGGCGTA
15MYB7065          ---------- ---------- ---------- ---------- ---------- ---------- -------GGT CTAGGATAGC ACGGAGGCTG CCAGGGCGTA
20MYB7065          ---------- ---------- ---------- ---------- ---------- ---------- -------GGT CTAGGATAGC ACGGAGGCTG CCAGGGCGTA
21MYB7065          ---------- ---------- ---------- ---------- ---------- ---------- -------GGT CTAGGATAGC ACGGAGGCTG CCAGGGCGTA
7MYB7065           ---------- ---------- ---------- ---------- ---------- ---------- -------GGT CTAGGATAGC ACGGAGGCTG CCAGGGCGTA
13MYB7065          ---------- ---------- ---------- ---------- ---------- ---------- -------GGT CTAGGATAGC ACGGAGGCTG CCAGGGCGTA
2MYB2094           ---------- ---------- ---------- ---------- ---------- ---------- -------GGT CTAGGATAGC ACGGAGGCTG CCAGGGCGTA
12MYB2094          ---------- ---------- ---------- ---------- ---------- ---------- -------GGT CTAGGATAGC ACGGAGGCTG CCAGGGCGCA
4MYB2094           ---------- ---------- ---------- ---------- ---------- ---------- -------GGT CTAGGATAGC ACGGAGGCTG CCAGGGCGTA
13MYB2094          ---------- ---------- ---------- ---------- ---------- ---------- -------GGT CTAGGATAGC ACGGAGGCTG CCAGGGCGTA
19MYB2094          ---------- ---------- ---------- ---------- ---------- ---------- -------GGT CTAGGATAGC ACGGAGGCTG CCAGGGCGTA
22MYB2094          ---------- ---------- ---------- ---------- ---------- ---------- -------GGT CTAGGATAGC ACGGAGGCTG CCAGGGCGTA
3MYB7065           ---------- ---------- ---------- ---------- ---------- ---------- -------GGT CTAGGATAGC ACGGAGGCTG CCAGGGCGTA
6MYB7065           ---------- ---------- ---------- ---------- ---------- ---------- -------GGT CTAGGATAGC ACGGAGGCTG CCGGGGCGTA
14MYB2094          ---------- ---------- ---------- ---------- ---------- ---------- -------GGT CTAGGATAGC ACGGAGGCTG CCAGGGCGTA
20MYB2094          ---------- ---------- ---------- ---------- ---------- ---------- -------GGT CTAGGATAGC ACGGAGGCTG CCAGGGCGTA
23MYB2094          ---------- ---------- ---------- ---------- ---------- ---------- -------GGT CTAGGATAGC ACGGAGGCTG CCAGGGCGTA
9MYB7065           ---------- ---------- ---------- ---------- ---------- ---------- -------GGT CTAGGATAGC ACGGAGGCTG CCAGGGCGCA
15MYB2094          ---------- ---------- ---------- ---------- ---------- ---------- -------GGT CTAGGATAGC ACGGAGGCTG CCAGGGCGCA
10MYB7065          ---------- ---------- ---------- ---------- ---------- ---------- -------GGT CTAGGATAGC ACGGAGGCTG CCAGGGCGCA
11MYG7065          ---------- ---------- ---------- ---------- ---------- ---------- -------GGT CTAGGATAGC ACGGAGGCTG CCAGGGCGCA
14MYB7065          ---------- ---------- ---------- ---------- ---------- ---------- -------GGT CTAGGATAGC ACGGAGGCTG CCAGGGCGCA
21MYB2094          ---------- ---------- ---------- ---------- ---------- ---------- -------GGT CTAGGATAGC ACGGAGGCTG CCAGGGCGCA
MYB_Mazz           ---------- ---------- ---------- ---------- ---------- ---------- -------GGT CTAGGATAGC ACGGAGGCTG CCAGGGCGTA
Clustal Consensus                                                                          *** ********** ********** ** ***** *

                           510        520        530        540        550        560        570        580        590        600
                   ....|....| ....|....| ....|....| ....|....| ....|....| ....|....| ....|....| ....|....| ....|....| ....|....|
16MYB2094          CAGACAACGA GATCAAGAAC TACTGGAGAA CACACATGAG AAAGAAAGCA CAGGAGAGGA AGATGAGCAT GTCATCACCT TCATCCTCAT CCTCCTCACT
24MYB2094          CAGACAACGA GATCAAGAAC TACTGGAGAA CACACATGAG AAAGAAAGCA CAGGAGAGGA AGATGAGCAT GTCATCACCT TCATCCTCAT CCTCCTCACT
8MYB2094           CAGACAACGA GATCAAGAAC TACTGGAGAA CACACATGAG AAAGAAAGCA CAGGAGAGGA AGATGAGCAT GTCATCACCT TCATCATCAT CCTCCTCACT
11MYB2094          CAGACAACGA GATCAAGAAC TACTGGAGAA CACACATGAG AAAGAAAGCA CAGGAGAGGA AGATGAGCAT GTCATCACCT TCATCATCAT CCTCCTCACT
3MYB2094           CAGACAACGA GATCAAGAAC TACTGGAGAA CACACATGAG AAAGAAAGCA CAGGAGAGGA AGATGAGCAT GTCATCACCT TCATCCTCAT CCTCCTCACT
17MYB2094          CAGACAACGA GATCAAGAAC TACTGGAGAA CACACATGAG AAAGAAAGCA CAGGAGAGGA AGATGAGCAT GTCATCACCT TCATCCTCAT CCTCCTCACT
1MYB2094           CAGACAACGA GATCAAGAAC TACTGGAGAA CACACATGAG AAAGAAAGCA CAGGAGAGGA AGATGAGCAT GTCATCACCT TCATCCTCAT CCTCCTCACT
8MYB7065           CAGACAACGA GATCAAGAAC TACTGGAGAA CACACATGAG AAAGAAAGCA CAGGAGAGGA AGATGAGCAT GTCATCACCT TCATCCTCAT CCTCCTCACT
2MYB7065           CAGACAACGA GATCAAGAAC TACTGGAGAA CACACATGAG AAAGAAAGCA CAGGAGAGGA AGATGAGCAT GTCATCACCT TCATCCTCAT CCTCCTCACT
17MYB7065          CAGACAACGA GATCAAGAAC TACTGGAGAA CACACATGAG AAAGAAAGCA CAGGAGAGGA AGATGAGCAT GTCATCACCT TCATCCTCAT CCTCCTCACT
22MYB7065          CAGACAACGA GATCAAGAAC TACTGGAGAA CACACATGAG AAAGAAAGCA CAGGAGAGGA AGATGAGCAT GTCATCACCT TCATCCTCAT CCTCCTCACT
15MYB7065          CAGACAACGA GATCAAGAAC TACTGGAGAA CACACATGAG AAAGAAAGCA CAGGAGAGGA AGATGAGCAT GTCATCACCT TCATCCTCAT CCTCCTCACT
20MYB7065          CAGACAACGA GATCAAGAAC TACTGGAGAA CACACATGAG AAAGAAAGCA CAGGAGAGGA AGATGAGCAT GTCATCACCT TCATCCTCAT CCTCCTCACT
21MYB7065          CAGACAACGA GATCAAGAAC TACTGGAGAA CACACATGAG AAAGAAAGCA CAGGAGAGGA AGATGAGCAT GTCATCACCT TCATCCTCAT CCTCCTCACT
7MYB7065           CAGACAACGA GATCAAGAAC TACTGGAGAA CACACATGAG AAAGAAAGCA CAGGAGAGGA AGATGAGCAT GTCATCACCT TCATCCTCAT CCTCCTCACT
13MYB7065          CAGACAACGA GATCAAGAAC TACTGGAGAA CACACATGAG AAAGAAAGCA CAGGAGAGGA AGATGAGCAT GTCATCACCT TCATCATCAT CCTCCTCACT
2MYB2094           CAGACAACGA GATCAAGAAC TACTGGAGAA CACACATGAG AAAGAAAGCA CAGGAGAGGA AGATGAGCAT GTCATCACCT TCATCCTCAT CCTCCTCACT
12MYB2094          CAGACAATGA GATCAAGAAC TACTGGAGAA CACACATGAG AAAGAAAGCT CAGGAGAGGA AGATGAGCAT GTCATCACCT TCATCATCAT CCTCCTCACT
4MYB2094           CAGACAACGA GATCAAGAAC TACTGGAGAA CACACATGAG AAAGAAAGCA CAGGAGAGGA AGATGAGCAT GTCATCACCT TCATCCTCAT CCTCCTCACT
13MYB2094          CAGACAACGA GATCAAGAAC TACTGGAGAA CACACATGAG AAAGAAAGCA CAGGAGAGGA AGATGAGCAT GTCATCACCT TCATCCTCAT CCTCCTCACT
19MYB2094          CAGACAACGA GATCAAGAAC TACTGGAGAA CACACATGAG AAAGAAAGCA CAGGAGAGGA AGATGAGCAT GTCATCACCT TCATCCTCAT CCTCCTCACT
22MYB2094          CAGACAACGA GATCAAGAAC TACTGGAGAA CACACATGAG AAAGAAAGCA CAGGAGAGGA AGATGAGCAT GTCATCACCT TCATCCTCAT CCTCCTCACT
3MYB7065           CAGACAACGA GATCAAGAAC TACTGGAGAA CACACATGAG AAAGAAAGCA CAGGAGAGGA AGATGAGCAT GTCATCACCT TCATCCTCAT CCTCCTCACT
6MYB7065           CAGACAACGA GATCAAGAAC TACTGGAGAA CACACATGAG AAAGAAAGCA CAGGAGAGGA AGATGAACAT TTCATCACCT TCATCCTCAT CCTCCTCACT
14MYB2094          CAGACAACGA GATCAAGAAC TACTGGAGAA CACACATGAG AAAGAAAGCA CAGGAGAGGA AGATGAGCAT GTCATCACCT TCATCCTCAT CCTCCTCACT
20MYB2094          CAGACAACGA GATCAAGAAC TACTGGAGAA CACACATGAG AAAGAAAGCA CAGGAGAGGA AGATGAGCAT GTCATCACCT TCATCCTCAT CCTCCTCACT
23MYB2094          CAGACAACGA GATCAAGAAC TACTGGAGAA CACACATGAG AAAGAAAGCA CAGGAGAGGA AGATGAGCAT GTCATCACCT TCATCCTCAT CCTCCTCACT
9MYB7065           CAGACAATGA GATCAAGAAC TACTGGAGAA CACACATGAG AAAGAAAGCT CAGGAGAGGA AGATGAGCAT GTCATCACCT TCATCCTCAT CCTCCTCACT
15MYB2094          CAGACAATGA GATCAAGAAC TACTGGAGAA CACACATGAG AAAGAAAGCT CAGGAGAGGA AGATGAGCAT GTCATCACCT TCATCCTCAT CCTCCTCACT
10MYB7065          CAGACAATGA GATCAAGAAC TACTGGAGAA CACACATGAG AAAGAAAGCT CAGGAGAGGA AGATGAGCAT GTCATCACCT TCATCCTCAT CCTCCTCACT
11MYG7065          CAGACAATGA GATCAAGAAC TACTGGAGAA CACACATGAG AAAGAAAGCT CAGGAGAGGA AGATGAGCAT GTCATCACCT TCATCCTCAT CCTCCTCACT
14MYB7065          CAGACAATGA GATCAAGAAC TACTGGAGAA CACACATGAG AAAGAAAGCT CAGGAGAGGA AGATGAGCAT GTCATCACCT TCATCCTCAT CCTCCTCACT
21MYB2094          CAGACAATGA GATCAAGAAC TACTGGAGAA CACACATGAG AAAGAAAGCT CAGGAGAGGA AGATGAGCAT GTCATCACCT TCATCCTCAT CCTCCTCACT
MYB_Mazz           CAGACAACGA GATCAAGAAC TACTGGAGAA CACACATGAG AAAGAAAGCA CAGGAGAGGA AGATGAGCAT GTCATCACCT TCATCCTCAT CCTCCTCACT
Clustal Consensus  ******* ** ********** ********** ********** *********  ********** ****** ***  ********* ***** **** **********
```

```
                           610        620        630        640        650        660        670        680        690        700
                    ....|....| ....|....| ....|....| ....|....| ....|....| ....|....| ....|....| ....|....| ....|....| ....|....|
16MYB2094           GACATACCAA GCCTGCCTTC TTGACACCAT TCCAGTCATT GGGATGGGCA GTGGTGACAC TCACAATGGT GGCTGCGTCA CCAGCGCCCT CGAGAACACA
24MYB2094           GACATACCAA GCCTGCCTTC TTGACACCAT TCCAGTCATT GGGATGGGCA GTGGTGACAC TCACAATGGT GGCTGCGTCA CCAGCGCCCT CGAGAACACA
8MYB2094            GACATACCAA GCCTGCCTTC TTGACACCAT TCCAGTCATT GGGATGGGCA GTGGTGACAC TCACAATGGT GGCTGCGTCA CCAGCGCCCT CGAGAACACA
11MYB2094           GACATACCAA GCCTGCCTTC TTGACACCAT TCCAGTCATT GGGATGGGCA GTGGTGACAC TCACAATGGT GGCTGCGTCA CCAGCGCCCT CGAGAACACA
3MYB2094            GACATACCAA GCCTGCCTTC TTGACACCAT TCCAGTCATT GGGATGGGCA GTGGTGACAC TCACAATGGT GGCTGCGTCA CCAGCGCCCT CGAGAACACA
17MYB2094           GACATACCAA GCCTGCCTTC TTGACACCAT TCCAGTCATT GGGATGGGCA GTGGTGACAC TCACAATGGT GGCTGCGTCA CCAGCGCCCT CGAGAACACA
1MYB2094            GACATACCAA GCCTGCCTTC TTGACACCAT TCCAGTCATT GGGATGGGCA GTGGTGACAC TCACAATGGT GGCTGCGTCA CCAGCGCCCT CGAGAACACA
8MYB7065            GACATACCAA GCCTGCCTTC TTGACACCAT TCCAGTCATT GGGATGGGCA GTGGTGACAC TCACAATGGT GGCTGCGTCA CCAGCGCCCT CGAGAACACA
2MYB7065            GACATACCAA GCCTGCCTTC TTGACACCAT TCCAGTCATT GGGATGGGCA GTGGTGACAC TCACAATGGT GGCTGTGTCA CCAGCGCCCT TGAGAACACA
17MYB7065           GACATACCAA GCCTGCCTTC TTGACACCAT TCCAGTCATT GGGATGGGCA GTGGTGACAC TCACAATGGT GGCTGTGTCA CCAGCGCCCT TGAGAACACA
22MYB7065           GACATACCAA GCCTGCCTTC TTGACACCAT TCCAGTCATT GGGATGGGCA GTGGTGACAC TCACAATGGT GGCTGTGTCA CCAGCGCCCT TGAGAACACA
15MYB7065           GACATACCAA GCCTGCCTTC TTGACACCAT TCCAGTCATT GGGATGGGCA GTGGTGACAC TCACAATGGT GGCTGTGTCA CCAGCGCCCT TGAGAACACA
20MYB7065           GACATACCAA GCCTGCCTTC TTGACACCAT TCCAGTCATT GGGATGGGCA GTGGTGACAC TCACAATGGT GGCTGTGTCA CCAGCGCCCT TGAGAACACA
21MYB7065           GACATACCAA GCCTGCCTTC TTGACACCAT TCCAGTCATT GGGATGGGCA GTGGTGACAC TCACAATGGT GGCTGTGTCA CCAGCGCCCT TGAGAACACA
7MYB7065            GACATACCAA GCCTGCCTTC TTGACACCAT TCCAGTCATT GGGATGGGCA GTGGTGACAC TCACAATGGT GGCTGCGTCA CCAGCGCCCT TGAGAACACA
13MYB7065           GACATACCAA GCCTGCCTTC TTGACACCAT TCCAGTCATT GGGATGGGCA GTGGTGACAC TCACAATGGT GGCTGCGTCA CCAGCGCCCT CGAGAACACA
2MYB2094            GACATACCAA GCCTGCCTTC TTGACACCGT TCGAGTCATT GGGATGGGCA GTGGTGACAC TCACAATGGT AGCTGCGTCA CCAGCGCCCT CGAGAACACA
12MYB2094           GACATACCAA GCCTGCCTTC TTGACACCAT TCCAGTCATT GGGATGGGCA GTGGTGACAC TCACAATGGT GGCTGCGTCA CCAGCGCCCT CGAGAACACA
4MYB2094            GACATACCAA GCCTGCCTTC TTGACACCAT TCCAGTCATT GGGATGGGCA GTGGTGACAC TCACAATGGT GGCTGCGTCA CCAGCGCCCT CGAGAACACA
13MYB2094           GACATACCAA GCCTGCCTTC TTGACACCAT TCCAGTCATT GGGATGGGCA GTGGTGACAC TCACAATGGT GGCTGCGTCA CCAGCGCCCT CGAGAACACA
19MYB2094           GACATACCAA GCCTGCCTTC TTGACACCAT TCCAGTCATT GGGATGGGCA GTGGTGACAC TCACAATGGT GGCTGCGTCA CCAGCGCCCT CGAGAACACA
22MYB2094           GACATACCAA GCCTGCCTTC TTGACACCAT TCCAGTCATT GGGATGGGCA GTGGTGACAC TCACAATGGT GGCTGCGTCA CCAGCGCCCT CGAGAACACA
3MYB7065            GACATACCAA GCCTGCCTTC TTGACACCAT TCCAGTCATT GGGATGGGCA GTGGTGACAC TCACAATGGT GGCTGCGTCA CCAGCGCCCT CGAGAACACA
6MYB7065            GACATACCAA GCCTGCCTTC TTGACACCGT TCCAGTCATT GGGATGGGCA GTGGTGACAC TCACAATGGT GGTTGCGTCA CCAGCGCCCT CGAGAACACA
14MYB2094           GACATACCAA GCCTGCCTTC TTGACACCAT TCCAGTCATT GGGATGGGCA GTGGTGACAC TCACAATGGT GGCTGCGTCA CCAGCGCCCT CGAGAACACA
20MYB2094           GACATACCAA GCCTGCCTTC TTGACACCGT TCCAGTCACT GGGATGGGCA GTGCCGACAC TCACAATGGT GGCTGCGTCA CCAGCGCCCT CGAGAACACA
23MYB2094           GACATACCAA GCCTGCCTTC TTGACACCAT TCCAGTCATT GGGATGGGCA GTGGTGACAC TCACAATGGT GGCTGCGTCA CCAGCGCCCT CGAGAATACA
9MYB7065            GACATACCAA GCCTGCCTTC TTGACACCGT TCCAGTCACT GGGATGGGCA GTGCCGACAC TCACAATGGT AGCTGTGTCA CCAGCGCCCT CGAGAACACA
15MYB2094           GACATACCAA GCCTGCCTTC TTGACACCGT TCCAGTCACT GGGATGGGCA GTGCCGACAC TCACAATGGT AGCTGTGTCA CCAGCGCCCT CGAGAACACA
10MYB7065           GACATACCAA GCCTGCCTTC TTGACACCGT TCCAGTCACT GGGATGGGCA GTGCCGACAC TCACAATGGT AGCTGTGTCA CCAGCGCCCT CGAGAACACA
11MYG7065           GACATACCAA GCCTGCCTTC TTGACACCGT TCCAGTCACT GGGATGGGCA GTGCCGACAC TCACAATGGT AGCTGTGTCA CCAGCGCCCT CGAGAACACA
14MYB7065           GACATACCAA GCCTGCCTTC TTGACACCGT TCCAGTCACT GGGATGGGCA GTGCCGACAC TCACAATGGT AGCTGTGTCA CCAGCGCCCT CGAGAACACA
21MYB2094           GACATACCAA GCCTGCCTTC TTGACACCGT TCCAGTCACT GGGATGGGCA GTGCCGACAC TCACAATGGT AGCTGTGTCA CCAGCGCCCT CGAGAACACA
MYB_Mazz            GACATACCAA GCCTGCCTTC TTGACACCAT TCCAGTCACT GGGATGGGCA GTGCCGACAC TCACAATGGT AGCTGTGTCA CCAGCGCCCT CGAGAACACA
Clustal Consensus   ********** ********** ******** * ** ***** * ********** ***  ***** **********  * ** **** **********  ***** ***

                           710        720        730        740        750        760        770        780        790        800
                    ....|....| ....|....| ....|....| ....|....| ....|....| ....|....| ....|....| ....|....| ....|....| ....|....|
16MYB2094           CAGAGTGTCA TGGATGGATA TCCCATAGAT CAGATATGGA ATGAGATTGA GGCACCGCAG GCACCTGCCT TGCTGGGTAT TGCTGAAGGG AAAGAGAAGA
24MYB2094           CAGAGTGTCA TGGATGGATA TCCCATAGAT CAGATATGGA ATGAGATTGA GGCACCGCAG GCACCTGCCT TGCTGGGTAT TGCTGAAGGG AAAGAGAAGA
8MYB2094            CAGAGTGTCA TGGATGGATA TCCCATAGAT CAGATATGGA ATGAGATTGA GGCACCGCAG GCACCTGCCT TGCTGGGTAT TGCTGAAGGG AAAGAGAAGA
11MYB2094           CAGAGTGTCA TGGATGGATA TCCCATAGAT CAGATATGGA ATGAGATTGA GGCACCGCAG GCACCTGCCT TGCTGGGTAT TGCTGAAGGG AAAGAGAAGA
3MYB2094            CAGAGTGTCA TGGATGGATA TCCCATAGAT CAGATATGGA ATGAGATTGA GGCACCGCAG GCACCTGCCT TGCTGGGTAT TGCTGAAGGG AAAGAGAAGA
17MYB2094           CAGAGTGTCA TGGATGGATA TCCCATAGAT CAGATATGGA ATGAGATTGA GGCACCGCAG GCACCTGCCT TGCTGGGTAT TGCTGAAGGG AAAGAGAAGA
1MYB2094            CAGAGTGTCA TGGATGGATA TCCCATAGAT CAGATATGGA ATGAGATTGA GGCACCGCAG GCACCTGCCT TGCTGGGTAT TGCTGAAGGG AAAGAGAAGA
8MYB7065            CAGAGTGTCA TGGATGGATA TCCCATAGAT CAGATATGGA ATGAGATTGA GGCACCGCAG GCACCTGCCT TGCTGGGTAT TGCTGAAGGG AAAGAGAAGA
2MYB7065            CATAGTGTCA TGGATGGATA TCCCATAGAT CAGATATGGA ATGAGATTGA GGCACCGCAG GCACCTGCCT TGCTGGGTAT TGCTGAAGGG AAAGAGAAGA
17MYB7065           CATAGTGTCA TGGATGGATA TCCCATAGAT CAGATATGGA ATGAGATTGA GGCACCGCAG GCACCTGCCT TGCTGGGTAT TGCTGAAGGG AAAGAGAAGA
22MYB7065           CATAGTGTCA TGGATGGATA TCCCATAGAT CAGATATGGA ATGAGATTGA GGCACCGCAG GCACCTGCCT TGCTGGGTAT TGCTGAAGGG AAAGAGAAGA
15MYB7065           CATAGTGTCA TGGATGGATA TCCCATAGAT CAGATATGGA ATGAGATTGA GGCACCGCAG GCACCTGCCT TGCTGGGTAT TGCTGAAGGG AAAGAGAAGA
20MYB7065           CATAGTGTCA TGGATGGATA TCCCATAGAT CAGATATGGA ATGAGATTGA GGCACCGCAG GCACCTGCCT TGCTGGGTAT TGCTGAAGGG AAAGAGAAGA
21MYB7065           CATAGTGTCA TGGATGGATA TCCCATAGAT CAGATATGGA ATGAGATTGA GGCACCGCAG GCACCTGCCT TGCTGGGTAT TGCTGAAGGG AAAGAGAAGA
7MYB7065            CAGAGTGTCA TGGATGGATA TCCCATAGAT CAGATATGGA ATGAGATTGA GGCACCGCAG GCACCTGCCT TGCTGGGTAT TGCTGAAGGG AAAGAGAAGA
13MYB7065           ---AGTGTCA TGGATGGATA TCCCATAGAT CAGATATGGA ATGAGATTGA GGCACCGCAG GCACCTGCCT TGCTGGGTAT TGCTGAAGGG AAAGAGAAGA
2MYB2094            CAGAGTGCCA TGGATGGATA TCCCATAGAT CAGATATGGA ATGAGATTGA GGCACCGCAG GCACCTGCCT TGCTGGGTAT TGCTGAAGGG AAAGAGAAGA
12MYB2094           ---AGTGTCA TGGATGGATA TCCCATAGAT CAGATATGGA ATGAGATTGA GGCACCGCAG GCACCTGCCT TGCTGGGTAT TGCTGAAGGG AAAGAGAAGA
4MYB2094            CAGAGTGTCA TGGATGGATA TCCCATAGAT CAGATATGGA ATGAGATTGA GGCACCGCAG GCACCTGCCT TGCTGGGTAT TGCTGAAGGG AAAGAGAAGA
13MYB2094           CAGAGTGTCA TGGATGGATA TCCCATAGAT CAGATATGGA ATGAGATTGA GGCACCGCAG GCACCTGCCT TGCTGGGTAT TGCTGAAGGG AAAGAGAAGA
19MYB2094           CAGAGTGTCA TGGATGGATA TCCCATAGAT CAGATATGGA ATGAGATTGA GGCACCGCAG GCACCTGCCT TGCTGGGTAT TGCTGAAGGG AAAGAGAAGA
22MYB2094           CAGAGTGTCA TGGATGGATA TCCCATAGAT CAGATATGGA ATGAGATTGA GGCACCGCAG GCACCTGCCT TGCTGGGTAT TGCTGAAGGG AAAGAGAAGA
3MYB7065            CAGAGTGTCA TGGATGGATA TCCCATAGAT CAGATATGGA ATGAGATTGA GGCACCGCAG GCACCTGCCT TTCTGGGTAT TGCTGAAGGG AAAGAGACGA
6MYB7065            CAGAGTGTCA TGGATGGATA TCCCATGGAT CAGATATGGA ATGAGATTGA GGCACCGCAG GCACCTGCCT TTCTGGGTAT TGCTGAAGGG AAAAAGACGA
14MYB2094           CAAAGTGCCA TGGATGGATA TCCCATGGAT CAGATATGGA AGGCGATTGA GGCACCGCAG GCACCTGCCT TGCTGGGTAT TGCTGAAGGG AAAGAGAAGA
20MYB2094           CAAAGTGCCA TGGATGGATA TCCCATGGAT CAGATATGGA AGGCGATTGA GGCACCGCAG GCACCTGCCT TTCTGGGTAT TGCTGAAGGG AAAGAGACGA
23MYB2094           CAAAGTGCCA TGGATGGATA TCCCATGGAT CAGATATGGA AGGCGATTGA GGCACCGCAG GCACCTGCCT TGCTGGGTAT TGCTGAACGA AAAGAGAAGA
9MYB7065            CAAAGTGCCA TGGATGGATA TCCCATGGAT CAGATATGGA AGGCGATTGA GGCACCGCAG GCACCTGCCT TGCTGGGTAT TGCTGAACGA AAAGAGAAGA
15MYB2094           CAAAGTGCCA TGGATGGATA TCCCATGGAT CAGATATGGA AGGCGATTGA GGCACCGCAG GCACCTGCCT TGCTGGGTAT TGCTGAACGA AAAGAGAAGA
10MYB7065           CAAAGTGCCA TGGATGGATA TCCCATGGAT CAGATATGGA AGGCGATTGA GGCACCGCAG GCACCTGCCT TGCTGGGTAT TGCTGAACGA AAAGAGAAGA
11MYG7065           CAAAGTGCCA TGGATGGATA TCCCATGGAT CAGATATGGA AGGCGATTGA GGCACCGCAG GCACCTGCCT TGCTGGGTAT TGCTGAACGA AAAGAGAAGA
14MYB7065           CAAAGTGCCA TGGATGGATA TCCCATGGAT CAGATATGGA AGGCGATTGA GGCACCGCAG GCACCTGCCT TGCTGGGTAT TGCTGAACGA AAAGAGAAGA
21MYB2094           CAAAGTGCCA TGGATGGATA TCCCATGGAT CAGATATGGA AGGCGATTGA GGCACCGCAG GCACCTGCCT TGCTGGGTAT TGCTGAACGA AAAGAGAAGA
MYB_Mazz            CAAAGTGCCA TGGATGGATA TCCCATGGAT CAGATATGGA ATGAGATTGA GGCACCGCAG GCACCTGCCT TGCTGGGTAT TGCTGAAGGG AAAGAGAAGA
Clustal Consensus      **** ** ********** ****** *** ********** * * ****** ********** ********** * ******** ******* *  *** *** **

                           810        820        830        840        850        860        870        880        890        900
                    ....|....| ....|....| ....|....| ....|....| ....|....| ....|....| ....|....| ....|....| ....|....| ....|....|
16MYB2094           CATGTAGCAG TATCCCCTGT CATCTACCAT CATCTGTTAT GTGGGACTAC AAATATCCTG AGGTGTTCTG GAAGATGGA- AGATAAAGAA ATCATGATGT
24MYB2094           CATGTAGCAG TATCCCCTGT CATCTACCAT CATCTGTTAT GTGGGACTAC AAATATCCTG AGGTGTTCTG GAAGATGGA- AGATAAAGAA ATCATGATGT
8MYB2094            CATGTAGCAG TATCCCCTGT CATCTACCAT CATCTGCTAT GTGGGACTAC AAATATCCTG AGGTATTCTG GAAGATGGA- AGATAAAGAA ATCATGA---
11MYB2094           CATGTAGCAG TATCCCCTGT CATCTACCAT CATCTGCTAT GTGGGACTAC AAATATCCTG AGGTATTCTG GAAGATGGA- AGATAAAGAA ATCATGA---
3MYB2094            CATGTAGCAG TATCCCCTGT CATCTACCAT CATCTGTTAT GTGGGACTAC AAATATCCTG AGGTATTCTG GAAGATGGA- AGATAAAGAA ATCATGA---
17MYB2094           CATGTAGCAG TATCCCCTGT CATCTACCAT CATCTGTTAT GTGGGACTAC AAATATCCTG AGGTATTCTG GAAGATGGA- AGATAAAGAA ATCATGA---
1MYB2094            CATGTAGCAG TATCCCCTGT CATCTACCAT CATCTGCTAT GTGGGACTAC AAATATCCTG AGGTATTCTG GAAGATGGA- AGATAAAGAA ATCATGA---
8MYB7065            CATGTAGCAG TATCCCCTGT CATCTACCAT CATCTGCTAT GTGGGACTAC AAATATCCTG AGGTATTCTG GAAGATGGA- AGATAAAGAA ATCATGATGT
2MYB7065            CATGTAGCAG TATCCCCTGT CATCTACCAT CATCTGCTAT GTGGGACTAC AAATATCCTG AGGTATTCTG GAAGATGGA- AGATAAAGAA ATCATGATGT
17MYB7065           CATGTAGCAG TATCCCCTGT CATCTACCAT CATNTGCTAT GTGGGACTAC AAATATCCTG AGGTATTCTG GAAGATGGA- AGATAAAGAA ATCATGATGT
22MYB7065           CATGTAGCAG TATCCCCTGT CATCTACCAT CATCTGNTAT GTGGGACTAC AAATATCCTG AGGTATTCTG GAAGATGGA- AGATAAAGAA ATCATGATGT
15MYB7065           CATGTAGCAG TATCCCCTGT CATCTACCAT CATCTGCTAT GTGGGACTAC AAATATCCTG AGGTATTCTG GAAGATGGA- AGATAAAGAA ATCATGATGT
20MYB7065           CATGTAGCAG TATCCCCTGT CATCTACCAT CATCTGCTAT GTGGGACTAC AAATATTCTG AGGTGTTCTG GAAGATGGA- AGATAAAGAA ATCATGATGT
21MYB7065           CATGTAGCAG TATCCCCTGT CATCTACCAT CATCTGCTAT GTGGGACTAC AAATATTCTG AGGTGTTCTG GAAGATGGA- AGATAAAGAA ATCATGATGT
7MYB7065            CATGTAGCAG TATCCCCTGT CATCTACCAT CATCTGCTAT GTGGGACTAC AAATATCCTG AGGTATTCTG GAAGATGGA- AGATAAAGAA ATCATGATGT
13MYB7065           CATGTAGCAG TATCCCCTGT CATCTACCAT CATGTGCTAT GTGGGACTAC AAATATCCTG AGGTATTCTG GAAGATGGA- AGATAAAGAA ATCATGATGT
2MYB2094            CATGTAGCAG TATCCCCTGT CATCTACCAT CATCTGTTAT GTGGGACTAC AAATATCCTG AGGTATTCTG GAAGATGGA- AGATAAAGAA ATCATGA---
12MYB2094           CATGTAGCAG TATCCCCTGT CATCTACCAT CATCTGCTAT GTGGGACTAC AAATATCCTG AGGTATTCTG GAAGATGGA- AGATAAAGAA ATCATGA---
4MYB2094            CATGTAGCAG TATCCCCTGT CATCTACCAT CATCTGTTAT GTGGGACTAC AAATATCCTG AGGTGTTCTG GAAGATGGA- AGATAAAGAA ATCATGA---
13MYB2094           CATGTAGCAG TATCCCCTGT CATCTACCAT CATCTGTTAT GTGGGACTAC AAATATCCTG AGGTGTTCTG GAAGATGGA- AGATAAAGAA ATCATGA---
19MYB2094           CATGTAGCAG TATCCCCTGT CATCTACCAT CATCTGTTAT GTGGGACTAC AAATATCCTG AGGTGTTCTG GAAGATGGA- AGATAAAGAA ATCATGA---
22MYB2094           CATGTAGCAG TATCCCCTGT CATCTACCAT CATCTGTTAT GTGGGACTAC AAATATCCTG AGGTGTTCTG GAAGATGGA- AGATAAAGAA ATCATGA---
3MYB7065            CATGCAGCAG TAACCCCTGT CATCTACCAT CATCTTCTAT GTGGGACTAC AAATATCCTG AGGTATTCTG GAAGATGGA- AGATAAAGAA ATCATGATGT
6MYB7065            CATGCAGCAG TAACCCCTGT CATCTACCAT CATCTTCTAT GTGGGACTAC AAATATCCTG AGGTATTCTG GAAGATGGA- AGATAAAGAA ATTATGATGT
14MYB2094           CATGTAGCAG TATCCCCTGT CATCTACCAT CATCTGCTAT GTGGGACTAC AAATATCCTG AGGTATTCTG GAAGATGGA- AGATAAAGAA ATCATGATGT
20MYB2094           CATGCAGCAG TATCCCCTGT CATCTACCAT CATCTTCTAT GTGGGACTAC AAATATCCTG AGGTGTTCTG GAAGATGGA- AGATAAAGAA ATCATGATGT
23MYB2094           CATGCAGCAG TATCCCCTGT CATCTACCAT CACCTGCTAT GTGGGACTAC AAATATTCTG AGGTGTTCTG GAAGATGGA- AGATAAAGAA ATCATGATGT
9MYB7065            CATGCAGCAG TATCCCCTGT CATCTACCAT CACCTGCTAT GTGGGACTAC AAATATTCTG AGGTGTTCTG GAAGATAAAG AAATCATGAT GTGA------
15MYB2094           CATGCAGCAG TATCCCCTGT CATCTACCAT CACCTGCTAT GTGGGACTAC AAATATTCTG AGGTGTTCTG GAAGATGGA- AGATAAAGAA ATCATGA---
10MYB7065           CATGCAGCAG TATCCCCTGT CATCTACCAT CACCTGCTAT GTGGGACTAC AAATATTCTG AGGTGTTCTG GAAGATGGA- AGATAAAGAA ATCATGATGT
11MYG7065           CATGCAGCAG TATCCCCTGT CATCTACCAT CACCTGCTAT GTGGGACTAC AAATATTCTG AGGTGTTCTG GAAGATGGA- AGATAAAGAA ATCATGATGT
14MYB7065           CATGCAGCAG TATCCCCTGT CATCTACCAT CACCTGCTAT GTGGGACTAC AAATATTCTG AGGTGTTCTG GAAGATGGA- AGATAAAGAA ATCATGATGT
21MYB2094           CATGCAGCAG TATCCCCTGT CATCTACCAT CACCTGCTAT GTGGGACTAC AAATATTCTG AGGTGTTCTG GAAGATGGA- AGATAAAGAA ATCATGATGT
MYB_Mazz            CATGTAGCAG TATCCCCTGT CATCTACCAT CATCTGTTAT GTGGGACTAC AAATATCCTG AGGTATTCTG GAAGATGGA- AGATAAAGAA ATCATGATGT
Clustal Consensus   **** ***** ** ******* ********** **  *  *** ********** ****** *** **** ***** ******  *  * ** * **   * *
```

```
                    ..
16MYB2094           GA
24MYB2094           GA
8MYB2094            --
11MYB2094           --
3MYB2094            --
17MYB2094           --
1MYB2094            --
8MYB7065            GA
2MYB7065            GA
17MYB7065           GA
22MYB7065           GA
15MYB7065           GA
20MYB7065           GA
21MYB7065           GA
7MYB7065            GA
13MYB7065           GA
2MYB2094            --
12MYB2094           --
4MYB2094            --
13MYB2094           --
19MYB2094           --
22MYB2094           --
3MYB7065            GA
6MYB7065            GA
14MYB2094           GA
20MYB2094           GA
23MYB2094           GA
9MYB7065            --
15MYB2094           --
10MYB7065           GA
11MYG7065           GA
14MYB7065           GA
21MYB2094           GA
MYB_Mazz            GA
Clustal Consensus
```
