# Supplementary figures and images for: Overexpression of ScMYBAS1 alternative splicing transcripts differentially impacts biomass accumulation and drought tolerance in rice transgenic plants
Source: PLoS One. 2018 Dec 5;13(12):e0207534. doi: 10.1371/journal.pone.0207534 (PMC6281192; doi:10.1371/journal.pone.0207534)

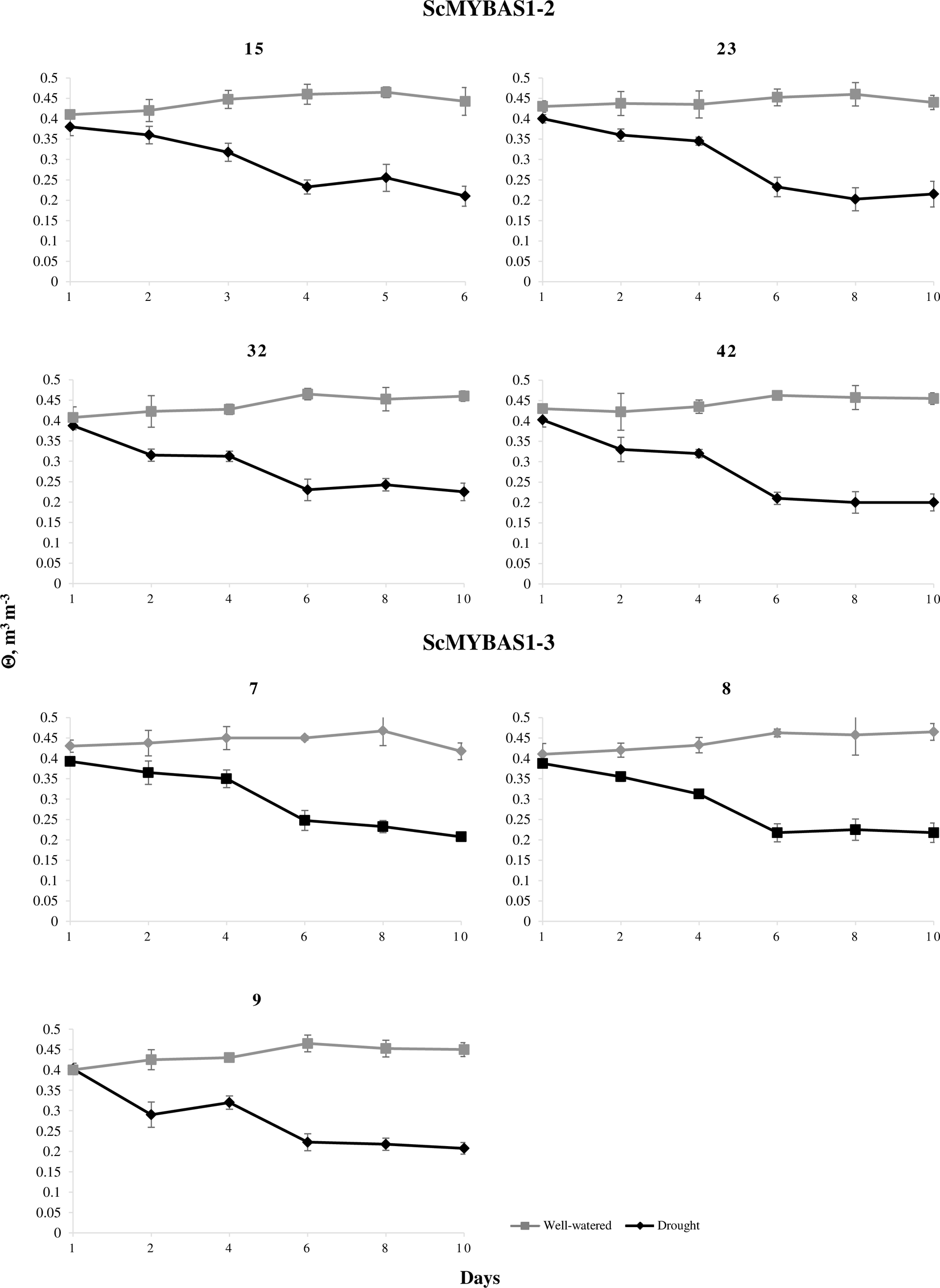

Supplement: S1 Fig — The values are mean ± SE (n = 4). (TIF) [file pone.0207534.s002.tif]
