# Supplementary material for: Overexpression of ScMYBAS1 alternative splicing transcripts differentially impacts biomass accumulation and drought tolerance in rice transgenic plants
Source: PLoS One. 2018 Dec 5;13(12):e0207534. doi: 10.1371/journal.pone.0207534 (PMC6281192; doi:10.1371/journal.pone.0207534)
Supplement: S1 Table — Protein sequences from monocots and A. thaliana: ScMYBAS1-2 (MH052202), ScMYBAS1-3 (MH052205), ScMYBAS1-4 (MH052203), ScMYBAS1-5 (MH052201), SbMYB23 (Sobic.005G224800.1), ZmMYB88 (AFW60113.1), OsMYBAS1-1 (Q53NK6-2), OsMYBAS1-3 (AK111626), AtMYB59-3 (AT5G59780.3) and AtMYB48-3 (AT3G46130.1). (PDF) [file pone.0207534.s003.pdf]

S1 Table

	AtMYB59-3	AtMYB48-3	OsMYBAS1-1	OSMYBAS1-3	ZmMYB88	ScMYBAS1-4	ScMYBAS1-3	SbMYB23	ScMYBAS1-2	ScMYBAS1-5
**AtMYB59-3**	100.00	80.60	44.44	55.96	56.67	46.90	58.10	58.10	48.28	33.01
**AtMYB48-3**	80.60	100.00	37.50	49.11	51.16	41.06	53.49	53.02	42.38	25.69
**OsMYBAS1-1**	44.44	37.50	100.00	100.00	72.22	70.37	71.60	72.84	72.22	64.17
**OSMYBAS1-3**	55.96	49.11	100.00	100.00	78.41	70.37	77.97	79.30	72.22	64.17
**ZmMYB88**	56.67	51.16	72.22	78.41	100.00	86.50	89.04	90.79	89.57	85.95
**ScMYBAS1-4**	46.90	41.06	70.37	70.37	86.50	100.00	89.57	92.02	92.02	90.91
**ScMYBAS1-3**	58.10	53.49	71.60	77.97	89.04	89.57	100.00	94.30	94.48	92.56
**SbMYB23**	58.10	53.02	72.84	79.30	90.79	92.02	94.30	100.00	95.09	94.21
**ScMYBAS1-2**	48.28	42.38	72.22	72.22	89.57	92.02	94.48	95.09	100.00	99.17
**ScMYBAS1-5**	33.01	25.69	64.17	64.17	85.95	90.91	92.56	94.21	99.17	100.00
